# Human-induced evolution caught in action: SNP-array reveals rapid amphi-atlantic spread of pesticide resistance in the salmon ecotoparasite *Lepeophtheirus salmonis*

**DOI:** 10.1186/1471-2164-15-937

**Published:** 2014-10-26

**Authors:** Francois Besnier, Matthew Kent, Rasmus Skern-Mauritzen, Sigbjørn Lien, Ketil Malde, Rolf B Edvardsen, Simon Taylor, Lina ER Ljungfeldt, Frank Nilsen, Kevin A Glover

**Affiliations:** Institute of Marine Research, Nordnes, Bergen, Norway; Centre for Integrative Genetics (CIGENE), Department of Animal and Aquacultural Sciences, Norwegian University of Life Sciences, 1432 Ås, Norway; Sea Lice Research Centre, Department of Biology, University of Bergen, Bergen, Norway

## Abstract

**Background:**

The salmon louse, Lepeophtheirus salmonis, is an ectoparasite of salmonids that causes huge economic losses in salmon farming, and has also been causatively linked with declines of wild salmonid populations. Lice control on farms is reliant upon a few groups of pesticides that have all shown time-limited efficiency due to resistance development. However, to date, this example of human-induced evolution is poorly documented at the population level due to the lack of molecular tools. As such, important evolutionary and management questions, linked to the development and dispersal of pesticide resistance in this parasite, remain unanswered. Here, we introduce the first Single Nucleotide Polymorphism (SNP) array for the salmon louse, which includes 6000 markers, and present a population genomic scan using this array on 576 lice from twelve farms distributed across the North Atlantic.

**Results:**

Our results support the hypothesis of a single panmictic population of lice in the Atlantic, and importantly, revealed very strong selective sweeps on linkage groups 1 and 5. These sweeps included candidate genes potentially connected to pesticide resistance. After genotyping a further 576 lice from 12 full sibling families, a genome-wide association analysis established a highly significant association between the major sweep on linkage group 5 and resistance to emamectin benzoate, the most widely used pesticide in salmonid aquaculture for more than a decade.

**Conclusions:**

The analysis of conserved haplotypes across samples from the Atlantic strongly suggests that emamectin benzoate resistance developed at a single source, and rapidly spread across the Atlantic within the period 1999 when the chemical was first introduced, to 2010 when samples for the present study were obtained. These results provide unique insights into the development and spread of pesticide resistance in the marine environment, and identify a small genomic region strongly linked to emamectin benzoate resistance. Finally, these results have highly significant implications for the way pesticide resistance is considered and managed within the aquaculture industry.

**Electronic supplementary material:**

The online version of this article (doi:10.1186/1471-2164-15-937) contains supplementary material, which is available to authorized users.

## Background

The salmon louse *Lepeophtheirus salmonis* (Krøyer, 1838) is an ectoparasitic copepod that feeds on the mucus, skin and blood of salmonid fishes in the marine environment. Through its feeding action, *L. salmonis* leads to stress [1, 2] and in severe untreated cases, open wounds and death of the host fish [3]. Within the Atlantic salmon (*Salmo salar* L.) and rainbow trout (*Onchorhynchus mykiss*) aquaculture industry that is primarily conducted in marine cages, *L. salmonis* infections cause highly significant economic losses [4, 5]. In addition, due to the rapid expansion of aquaculture, the evolutionary dynamics of the relationship between *L. salmonis* and its hosts has changed [6]. Furthermore, there is evidence that *L. salmonis* is associated with declines of wild salmonid populations [5, 7, 8], mortality in the marine environment [9, 10], and modifications of life history traits such as the age of return from the sea [11]. As a consequence, lice control regimes have been put into place to reduce the level of sea lice infestation in marine farms.

A range of integrated methods are currently employed or being developed to control infestations of sea lice on salmonids in marine farms [12–16]. However, the industry continues to rely heavily upon the use of a few certified pesticides to remove lice from infested fish. This is not considered as a sustainable approach [17, 18] due to a high risk of resistance development. Currently, over 500 arthropod pest-species are documented as having developed resistance to pesticides [19], and *L. salmonis*, as for arthropods with short generation times and high reproduction rates, has a strong capacity to adapt to new environmental constraints. Indeed, *L. salmonis* is known for developing resistance to chemical treatments [19–22], and loss of efficiency has been reported for a number of chemicals, for example organophosphates [23] and pyrethroids [24], and no new chemicals have been introduced since 1999 in Norway when SLICE® came on the market. During the past ten years, the main pesticide used for treating lice infestations in the North-Atlantic has been SLICE*®* emamectin benzoate (EMB) [25]. Emamectin benzoate is an avermectin where the target is gamma-aminobutyrate (GABA) and glutamate-gated chloride channels (GABA-Cls and Glu-Cls) [26]. However, the target site of this chemical in *L. salmonis* is not confirmed, and over reliance on the product has also led to the development of reduced sensitivity and resistance to EMB in sea lice populations [27–31].

Despite thorough efforts to unravel the mechanisms behind the development of pesticide resistance in *L. salmonis* [20, 21, 32, 33], little is known about the genetics and genomics of this species at the population level. Whether resistance to pesticides has developed in *L. salmonis* in multiple farms and regions in parallel, or has developed in one location and spread thereafter, is completely unknown. Development of resistance in a pest population submitted to intense control is however expected to significantly alter the pattern of allelic frequencies around the positively selected genomic region [34, 35]. In the case of the salmon louse, addressing this issue is not only essential for the development of management guidelines, it also has a high level of relevance to questions regarding the evolutionary relationships among lice throughout the Atlantic. This is because the question of population division remains a subject that has not been resolved, with previous studies being hampered by either a limited number of samples [36–38], or limited numbers of polymorphic loci [39, 40].

**Figure 1:**
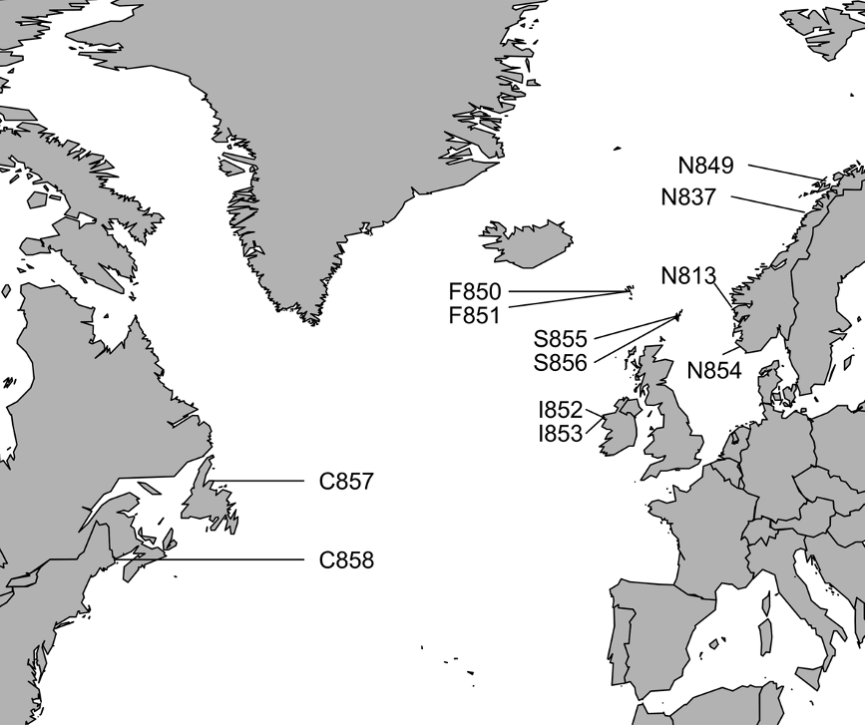
***Lepeophtheirus salmonis*** **sample locations throughout the Atlantic.** The letter in front of a sample number indicates the source: C = Canada, F = Faroe Islands, I = Ireland, N = Norway, S = Shetland.

In order to address pesticide resistance development and dispersal, in addition to evolutionary connectivity among *L. salmonis* throughout the North Atlantic, we developed a 6 k SNP array, and used it to genotype geographically distinct samples (Figure 1). The primary aim of the present study was to address the following questions: 1- Does *L. salmonis* display any population structure throughout the North Atlantic? 2- Is it possible to detect contemporary evolution linked with pesticide resistance development in *L. salmonis*? 3- Is it possible to estimate how fast and how far advantageous mutations, such as those conveying pesticide resistance, can be spread throughout the Atlantic?

## Results

### SNP array

A 6 k SNP array was produced for *L. salmonis*, based on an initial set of 640,000 polymorphic sites detected in five pooled samples of *L. salmonis* originating from five regions of the North Atlantic. Among the 6000 SNPs selected for inclusion on the array, 5080 were selected to cover the genome with an average interval of 100 kb (“genome distributed”), 174 were selected to cover a few larger scaffolds with average inter-SNP interval of 10 kb (“LD”), and 190 were selected based upon being located in or close to putative genes, based on matches to Expressed Sequence Tag (“ESTs”) regions. In addition, 556 SNPs were selected to provide a potential diagnostic tool to differentiate between populations throughout the Atlantic (“diagnostic”).

After production, 5540 of the original 6000 SNPs (92.3%) were successfully converted into on-chip assays including 508 diagnostic (91.2%), 170 EST (89.5%), 159 LD (91.4%), and 4703 (92.6%) genome distributed.

The SNP array was then used to genotype DNA collected from 576 individuals sampled from 12 commercial farms situated in the North Atlantic (Figure 1). After automatic clustering and manual validation, the average sample call rate was 99.5%, as was the average SNP call rate. Of the 5540 from 6000 SNPs, 121 (2.2%) were monomorphic, 209 (3.8%) displayed evidence of secondary SNPs within the flanking sequence making genotype calls unreliable, and 110 (2.0%) assays failed. Data from these three SNP categories, totaling 440 assays, was excluded from the present study. The remaining 5100 SNPs were further categorized as showing typical biallelic distribution with good cluster separation (SNP; 4666, 84.2%), showing a tight but distinct cluster distribution between allelotypes and requiring manual validation (n = 68, 1.2%), and 366 (6.6%) presenting some evidence of atypical clustering and also demanding manual checking. In a parallel project to the present study, a genetic linkage map was constructed (unpublished data) which included 5091 of the successfully genotyped SNPs. Data from these 5091 SNPs are included in the present study (Additional file 1).

### Population structure

**Figure 2:**
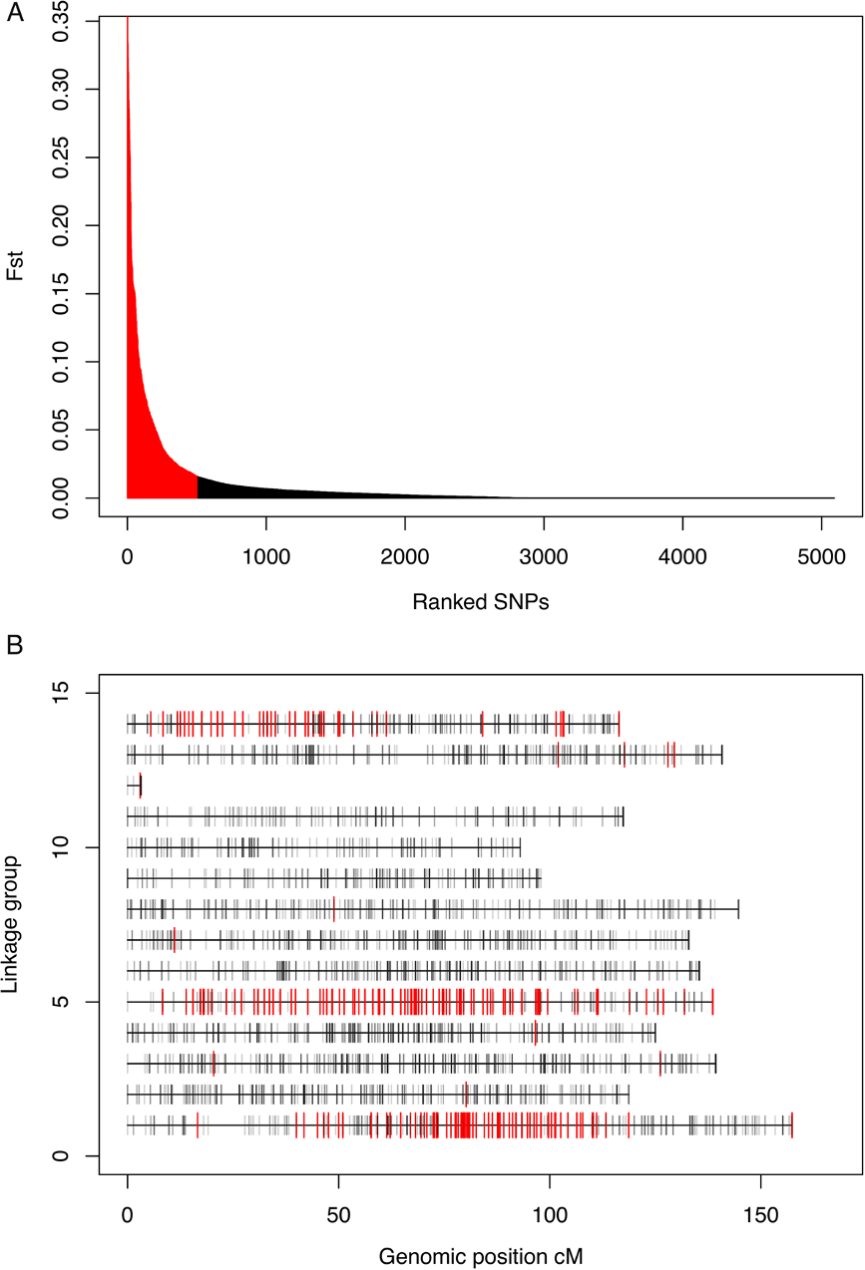
**Distribution of SNPs relative to global inter–region F**_**ST**_ **value. A**. barplot where SNPs are ranked on the x axis in decreasing order of F_ST_ value. The highest 10% values are represented with red bars. **B**. Distribution of the SNPs position on the linkage map. linkage groups 1 to 14 are represented as horizontal bars with length proportional to recombination frequencies in cM. Each SNP is represented as a vertical grey bar. SNPs displaying the highest 10% F_ST_ values are represented with red bars.

To infer the genetic distance among populations, F statistics were calculated, both pair wise, for all pairs of sampling sites, and globally, to assess the collective genetic distance among all regions. The value of global F_ST_ among regions ranged between 0 and 0.36 per SNP, with an average F_ST_ = 0.01 ± 0.04 across all SNPs. Only 2% of the SNPs displayed F_ST_ values larger than 0.1, and 90% displayed F_ST_ values smaller than 0.02 (Figure 2A). Thus, North Atlantic populations of *L. salmonis* displayed low genetic differentiation to each other. Significantly, SNPs displaying the highest genetic distances among samples were not evenly distributed throughout the genome. These markers were grouped on three genomic regions of linkage groups (LG)1, 5 and 14 (Figure 2B). When comparing pair-wise genetic distances and geographical distances, no SNP displayed significant correlation between geographical distance and pair wise F_ST_.

In addition to F statistics, the genotypes of all 576 individuals on the neutral markers were analyzed with Bayesian clustering implemented in STRUCTURE [41], and with the R package ADEgenet [42] using the “find.clusters” function. With those two approaches, the parameter K, which stands for the number of populations or clusters in the dataset, was tested for values from K = 1 to 12. Both log likelihood of the data calculated by STRUCTURE and the Bayesian Information Criterion (BIC) calculated from ADEgenet reached a minimum value (-351000 and 3790 respectively) for K = 1 as the number of clusters in the data. The two approaches gave consistent results, and indicated that *L. salmonis* consists of one single population throughout the North Atlantic.

### Outlier detection

**Figure 3:**
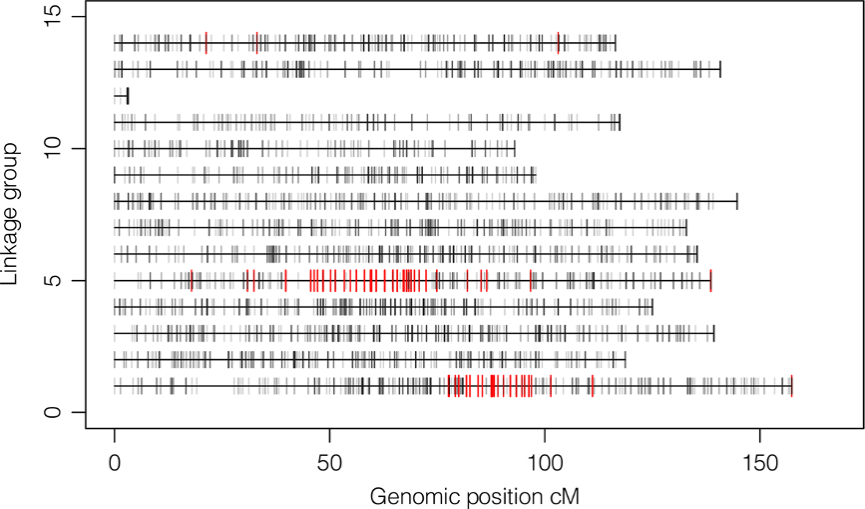
**Distribution of SNPs under positive selection on the linkage map.** Linkage groups from 1 to 14 are represented as horizontal bars with length proportional to recombination frequencies in cM. Each SNP is represented as a vertical grey bar. SNPs under positive selection are colored in red.

To infer the presence of SNPs under positive selection in the studied sets of samples, the genotypes of all individuals from twelve sampling sites were scanned for outlier markers using five different approaches. While the five scans gave slightly different results (Table 1), 167 markers were detected as being under positive selection in three or more scans out of five, and 4810 SNPs were detected as neutral markers in all five scans. The *L. salmonis* linkage map revealed that most markers under positive selection were located on two genomic regions: one region on LG1 between 80 and 100 cM (centimorgan), and a second region on LG5 between 45 and 75 cM (Figure 3). Of the 167 markers identified as being under positive selection, 42 were located on LG1, 121 were located on LG5, and 3 were located on LG14. All other linkage groups were exempt from outlier SNPs under selection.

**Table 1:** **Correlation (r** ^**2**^ **) between five genome scans for outlier markers**

	LOSITAN 6pop	LOSITAN 12pop	BayeScan 6pop	BayeScan 12pop	Arlequin F _CT_12pop
LOSITAN 6pop	1	0.70	0.55	0.53	0.72
LOSITAN 12pop		1	0.78	0.76	0.83
BayeScan 6pop			1	0.93	0.71
BayeScan 12pop				1	0.69
Arlequin F_CT_ 12pop					1

### Population demography

**Figure 4:**
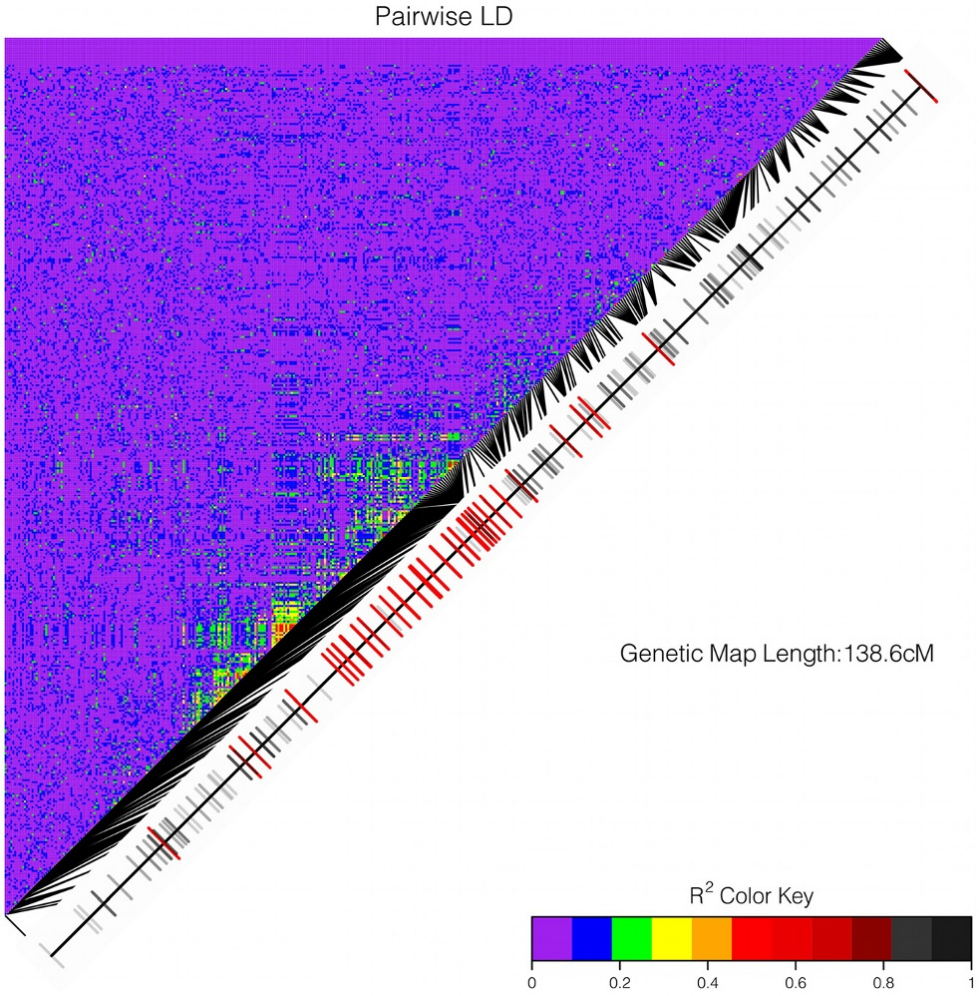
**Pairwise linkage disequilibrium among SNPs on linkage group 5 in sample I852 (Ireland).** Linkage map with SNP position in cM is represented on the diagonal of the LD matrix. SNPs under positive selection are colored in red. Black segments connect the SNP positions on the linkage map to the SNP positions on the LD matrix.

Despite the pattern of Linkage disequilibrium (LD) being constant over most of the genome, we observed local disturbance of LD in most populations on LG1 and LG5, in the same regions where SNPs under positive selection were clustered (Figure 4). LD was tested for each marker pair, and the average correlation coefficient between pairs of loci was r^2^ = 0.02. With the exception of two genomic regions under selection, this value of LD was constant across the genome, and only regions covered by dense SNPs, selected for short interval of 10 kb displayed higher LD, with an average of r^2^ = 0.35. The effective population size (Ne) was estimated independently in each sampling site from a sample of 100 unlinked SNPs. The estimated Ne ranged between 333 and infinity (Table 2), with Canada displaying the smallest population sizes, between Ne = 333 and Ne = 682, whereas South Norwegian populations seemed to have the largest effective size with an infinite estimate. An infinite estimate is simply interpreted as a large population [43].

**Table 2:** **Estimated effective population size from each sampling site**

	Estimated Ne	Lower 95% CI	Upper 95% CI
N854	inf	676	inf
N813	inf	584	inf
N849	2296	320	inf
N837	3140	333	inf
F850	inf	632	inf
F851	880	254	inf
S855	557	218	inf
S856	4277	339	inf
I852	761	246	Inf
I853	1217	280	inf
C857	333	171	2454
C858	682	237	inf

### Genomic regions affected by directional selection

**Figure 5:**
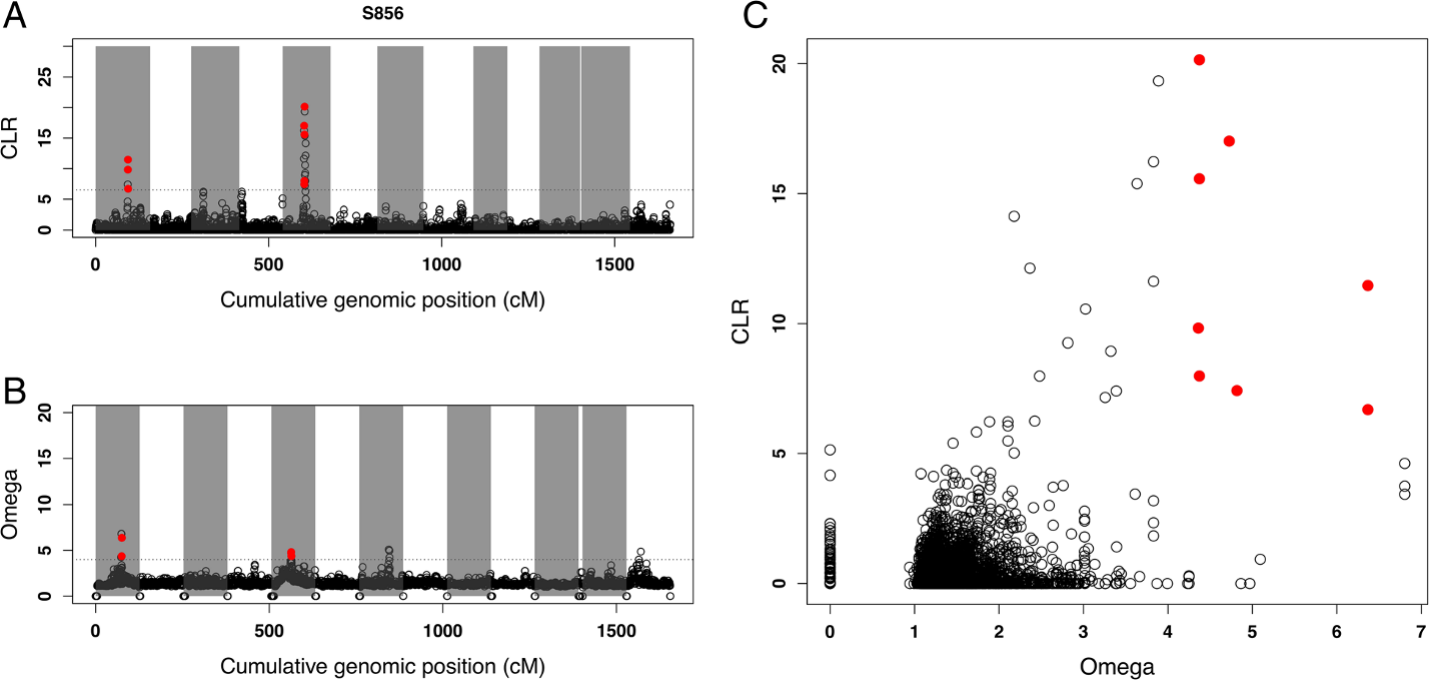
**Genome scan of Shetland (S856) sample for selective sweeps. A** and **B** represent respectively CLR calculated by SweeD and Omega statistic calculated by OmegaPlus on the y-axis, and the genome position in cM on the x-axis. Alternating grey and white areas separates linkage groups. **C** represents the joint plot of CLR and omega statistic. SNPs detected as outlier (p < 0.05) by both methods are in red.

The clustering of SNPs under positive selection on two genomic regions, on linkage groups 1 and 5, in addition with the local disturbance of LD pattern on these same regions, suggested that there might have been selective sweeps of beneficial alleles in the studied populations. The presence of selective sweeps was tested by scanning the genome for i) regions of reduced variability and ii) local pattern of high linkage disequilibrium. This was performed with two statistics, respectively CLR (Composite Likelihood Ratio) [44] and Omega [43]. Both CLR and Omega reported SNPs displaying a significant (p <0.05) pattern of selective sweep along the genome. A selective sweep was identified in a genomic region of LG1 by both CLR and Omega in sampling site S856 (Figure 5A and B), and by Omega only in sites F850, F851, S855, and I852 (Table 3). A second selective sweep was identified in a region of LG5 by both CLR and Omega in sampling site S856 (Figure 5A and B), I852, I853 and by Omega only in sites N849 and N837 (Table 3).

In sample S856, 8 SNPs (3 SNPs in LG1 and 5 SNPs in LG5) were identified as outliers (p < 0.05) by both CLR and Omega statistic (Figure 5C).

**Figure 6:**
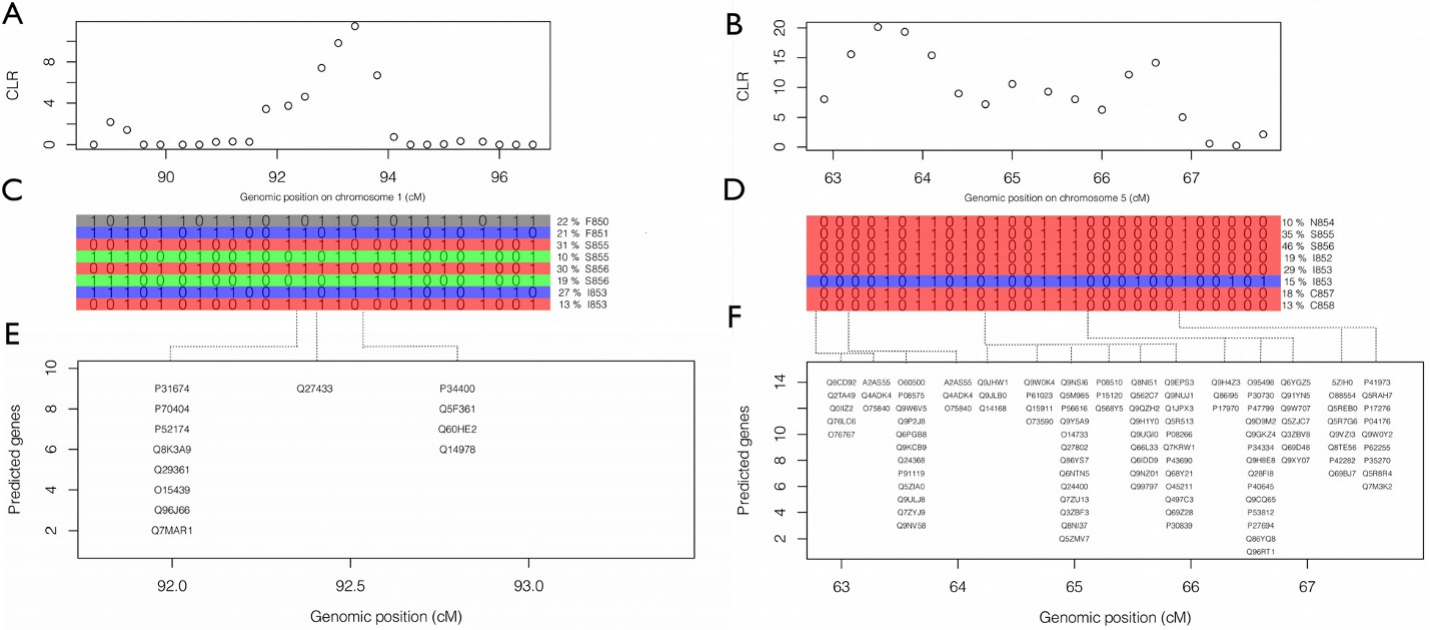
**Close-up of CLR scans for selective sweeps on linkage group 1 and linkage group 5 in S856 sample. A** and **B**: Close up of CLR scan for selective sweep on linkage group 1 and 5 in S856 sample. **C** and **D**: haplotypes present with frequency >10% in the same two genomic regions, and individualized by color. Sampling sites are reported together with haplotype frequency in percent within sampling site. **E** and **F**: predicted genes under the same two genomic regions.

The selective sweep on LG1 covered a genomic region of 2 cM, (1 cM ≈ 1% recombination per generation) i.e., a genomic region in which chromosome recombination would occur in 2% on the individuals in each generation, while the sweep on LG2 covered a 5 cM region. (Figure 6A and B).

Reconstruction of the marker phases under the selective sweep regions revealed that some haplotypes were present in high frequency (f >10%) in samples from all regions (Figure 6C and D). Moreover, we observed that the same haplotype sequence was found in high frequency, in sampling sites that are geographically distant by thousands of kilometers. For example, on LG5, between 63 and 67 cM, the same haplotype “00001011010100111000000100000” was shared with a frequency from 10%, and up to 46% in most regions from Norway to Canada. To test whether this pattern was specific for this region displaying a selective sweep, we also reconstructed haplotypes of similar size in 10 neutral genomic regions in all sampling sites. Among all neutral genomic regions, and all sampling sites, the most frequent haplotype was present in 3% of the population. By comparing the frequency of the most common haplotype in neutral genomic regions versus regions displaying selective sweeps, we show here that the observed haplotype frequency under region displaying selective sweeps is not likely to happen by chance in a neutral region of the genome.

**Table 3:** **Detection of selective sweeps from the two summary statistics, CLR and Omega, on the twelve sampling sites**

Sample	Chr1 (92–94 cM)		Chr5 (63–67 cM)	
	CLR	Omega	CLR	Omega
N854	NS	NS	NS	NS
N813	NS	P < 0.05	NS	NS
N849	NS	NS	NS	P < 0.05
N837	NS	NS	NS	P < 0.05
F850	NS	P < 0.05	NS	NS
F851	NS	P < 0.05	NS	NS
S855	NS	P < 0.05	NS	NS
S856	P < 0.05	P < 0.05	P < 0.05	P < 0.05
I852	NS	P < 0.05	P < 0.05	P < 0.05
I853	NS	NS	P < 0.05	P < 0.05
C857	NS	NS	NS	NS
C858	NS	NS	NS	NS

### Gene annotation

Matches for coding DNA were found in the genomic regions under positive selection, in 3 and 15 scaffolds on LG1 and LG5 respectively. Those scaffolds were 440 Kb length on average, and covered together 1.7 Mb on LG1 and 6.2 Mb on LG5. The number of predicted genes was respectively 13 and 110 in the two regions (Figure 6E and F, Additional file 2: Table S1). Functional annotation from BLAST hits revealed that some of these genes could play a role in drug resistance mechanisms. This was the case for Q96J66 an ATP-binding cassette (ABC) transporter protein predicted near marker c8501.30679 on LG1, Q28FI8, a cytochrome b5 domain-containing protein predicted near marker c8125.67718 on LG 5, or P17970 and P08510, two Potassium voltage-gated channel proteins predicted on LG 5 near markers c10059.23896, and c15581.45069 respectively (Additional file 2: Table S1, and Additional file 3: Table S2).

Despite showing a weaker signals for selective sweep than the reported regions on LG1 and LG5, one genomic region on LG14 did gather an important number of SNPs with high F_ST_ values (Figure 2B). Moreover, a portion of LG14 between 28 and 31 cM showed significant signal for selective sweeps in the Faeroe Island and Shetland samples (Additional file 4: Figure S1). This region was annotated following the same protocol as for the two previous regions. Matches for coding DNA were found for 6 scaffolds within which 19 genes were predicted. Among those, 1 gene (Q86GC8) coded for acetycholinesterase (Additional file 2: Table S1). This is the target for organophosphates and could be involved in resistance as a knock down mutation.

### Association between selective sweep regions and drug resistance

**Figure 7:**
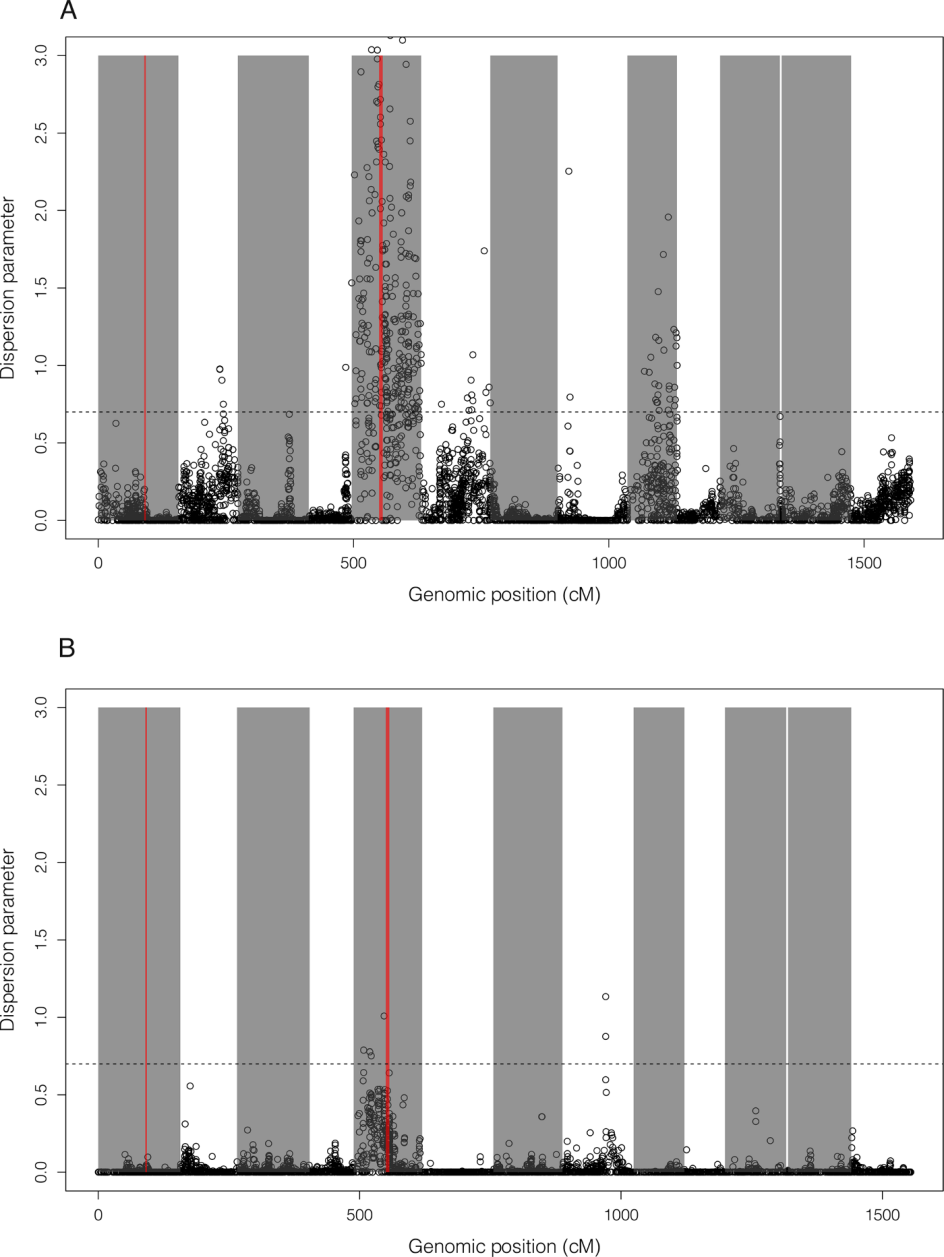
**Genome scan for association between 3 consecutive SNPs haplotypes and resistance to EMB, in the F2 dataset (A), and F1 dataset (B).** Cumulative genomic position is represented on the X axis with alternating grey and white areas to delimitate linkage groups. Dispersion parameter of the random genetic effect is reported on the Y axis. Areas corresponding to selective sweeps are colored in red. The 5% significance threshold is represented as horizontal dashed line.

A possible link between the detected selective sweeps and pesticide resistance was investigated for EMB, the most commonly used pesticide for reducing infestations of *L. salmonis* on farmed salmonids in the Atlantic in the period 2000 to 2010, which is the time-line when samples for this study were collected from commercial farms. To achieve this, a further set of 576 individuals representing 12 full sibling families displaying differential sensitivity to EMB [31] was genotyped on the SNP array. The dataset was split into two separate groups of individuals; 5 F1 families originated from crosses between resistant and susceptible strains, and 7 F2 families were crosses between parents of undetermined susceptibility to the chemical. The offspring of each family were exposed to EMB, and the status of each individual was reported as dead or alive after exposure [31]. Association between haplotypes at three consecutive markers and EMB sensitivity was investigated by fitting a Hierarchical Generalized Linear Model with the R package HGLM [45]. The dispersion parameter for the random genetic effect (Figure 7A and B) demonstrates that a broad region around the selective sweep on linkage group 5 had a strong and significant (p < 0.05) contribution to the variation in EMB resistance. In addition to LG5, 4 other genomic regions displayed significant association with EMB resistance in the F2 dataset, on linkage groups 2, 6, 8 and 9 (Figure 7A). In the F1 dataset, significant associations were found only on linkage groups 5 and 8, with generally lower values than in the F2 dataset (Figure 7B).

### Extrapolation of selection time frame

In LG5, we detected conserved haplotypes on a 5 cM region, which corresponds to a genomic region where chromosome recombination would occur with 5% frequency every generation i.e. once every 20 meiosis. Assuming the most drastic selection scenario where the frequency of the advantageous variant is nearly 100% in the population after a punctual selection event (exposure to chemical treatment of lice infestation), the frequency of a 5 cM haplotype associated to this mutation would decrease by 5% every generation following selection. A lapse of 15 generations would thus be needed to observe a decrease of haplotype frequency to 46% (as in sample S856) and, similarly, 43 generations to decrease to 10%.

## Discussion

### Population structure

The population genomic structure of *L. salmonis* throughout the Atlantic ocean was investigated using F statistics [46], principal component [42], and Bayesian clustering analysis [41] on genetic data from >5000 SNPs and 12 sets of samples from six regions. These analyses did not reveal any geographical genetic structure of *L. salmonis* throughout the North Atlantic. The overall global F_ST_ value was very low, with 98% of the markers displaying F_ST_ lower than 0.1. Furthermore, no significant correlation between genetic distance, measured as F_ST_, and geographical distance, were detected. Both Bayesian clustering and principal component analyses suggested K = 1 as the most likely number of clusters in the samples. Previous studies of population genetic structure of *L. salmonis* throughout the Atlantic have revealed contrasting results, ranging from highly significant genetic differentiation among nearby farms, to no or little structure throughout the entire North Atlantic. However, many of these earlier studies have been hindered by technical challenges, low sample sizes, low numbers of polymorphic markers, or combinations of these challenges [36–40]. Nevertheless, the most rigorous of the earlier studies, using data from four mitochondrial DNA (mtDNA) genes [37], and microsatellite markers on large numbers of samples [39, 40] have all revealed statistically weak or non-significant genetic differentiation throughout the Atlantic. The results of these former studies together with the present population genomic analysis strongly suggest that *L. salmonis* is characterized by a single panmictic population throughout the North Atlantic. The most likely mechanism for the absence of isolation by distance in such a large geographical area is transport by the salmon host. Wild Atlantic salmon migrating from rivers located on both sides of the Atlantic are known to mix on distant feeding grounds in the high seas of the Atlantic ocean [47]. Seaward migrating smolts originating from these American and European rivers may often be infected by salmon lice in their respective coastal zones, and thus transport these to the offshore feeding grounds where cross-infection of salmon from other geographic regions can occur. Such a mechanism would explain the lack of isolation by distance reported here. This hypothesis is further supported by the short time frame needed for an advantageous mutation to spread in the whole North Atlantic as reported here.

### Population demography

In several sites, the estimate (Nê) of the effective population size was infinite. This is usually interpreted as a signal of a large population where genetic drift is unlikely to be of major influence on the evolutionary properties of the population. The estimate of infinity occurs because the method implemented in LDNe [48], as other methods for estimating Ne, depends on a signal that is a function of 1/Ne [43]. As a consequence, the precision in estimating Ne is good for small populations, but the method has difficulties in distinguishing between large and infinite populations.

Among the 12 sampling sites studied here, Nê varied between 333 and infinity. This estimation remains imprecise, but is sufficient to suggest that a high proportion of the population inheriting large haplotype segments from a common ancestor, as observed in this study, is unlikely to happen by chance.

### Genomic regions affected by directional selection

Selective sweeps are the signature of a strong selection event where the incremented frequency of a selected locus in a population is accompanied by hitchhiking of the flanking genomic regions [34, 35]. The size of a genomic region affected by hitchhiking depends upon the recombination rate and the strength of the selection. In cases of high recombination rate and weak selection, the area may be small and thus, difficult to detect with a moderate density of molecular markers. In the literature, occurrence of selective sweeps have been reported in extensively studied organisms for which molecular resources are highly available, such as human [49, 50], or *Drosophila* [51–53], and in domestic species for which directional selection was very strong in the recent history [54–57]. Another example of reported selective sweeps is the development of chemical resistance in pest species or human disease vectors that are subject to intense population control [58–62]. In this latter example, the selection pressure is often very strong and led the selected variant to colonize the population in a short period of time. Such examples of human-induced evolution appear to be easier to detect as they happen on a contemporary time scale where recombination has not had the chance to erode the haplotype segments in the hitchhiking regions. In such cases, selective sweeps may be detectable with a medium density of molecular markers. This last case scenario has remarkable similarities with the results presented here, which re-enforces the main hypothesis of selection driven by drug resistance.

Combining two independent methods increases the power of sweep detection [63]. Here, we focused on genomic regions where selective sweeps were reported using both the CLR and Omega methods. Using these criteria, two selective sweeps were identified on linkage groups 1 and 5 respectively. These two genomic regions were already identified to contain a large number of the outlier markers identified as under positive selection, and displayed local disturbance in LD. It is thus highly likely that these two regions contain one or several advantageous mutations that were positively selected in *L. salmonis*.

The two selective sweep regions contained large haplotypes with frequencies higher that 10% within several sampling sites across the entire North Atlantic. Because the selection of an advantageous mutation can be accompanied by genetic hitchhiking [34], which creates LD patterns around the target of positive selection [64, 65], it is not surprising that haplotypes under the regions where selective sweep were detected are present in high frequencies in each sample. This is an expected consequence of the stronger LD observed in those regions. Interestingly, identical haplotypes were also frequent in geographically distant regions. The presence of selective sweeps coinciding with large haplotype segments conserved across distant localities strongly suggests that a de-novo mutation appeared between 10 to 40 generations before the sample used in the present study were collected. The causative mutation is likely to have occurred in one single geographical site, before being spread to the other regions rather than originating from multiple and independent selection events. This consolidates the conclusion above that *L. salmonis* is represented by a single panmictic population throughout the North Atlantic, and importantly, demonstrates that alleles conveying resistance to pesticides may be quickly spread over very large areas in the marine environment. This unique documentation of genetic resistance dispersal in the ocean reveals a major challenge for the management of resistance development within the aquaculture industry in the entire Atlantic. Because resistance can quickly spread across the ocean, management of pesticide resistance in this parasite needs to be addressed on an ocean-wide level rather than on a regional level. Moreover, due to the heterogeneous structure of the landscape where *L. salmonis i*s evolving, with patches of high host density in salmon farms and coastal areas, and large areas of low host density in the offshore regions, standard population genetic models are likely to produce biased estimates of the evolutionary dynamics of this organism [66].

### Gene annotation

In order to better identify the possible targets of positive selection detected on linkage groups 1 and 5, the two genomic regions were annotated to identify sequences of coding DNA together with the possible function of such sequences. The number of predicted genes on the scaffolds situated in the genomic regions under selection was 13 and 110 on linkage groups 1 and 5 respectively. Functional annotation was obtained from BLAST hits [67]. In most cases, BLAST from a given sequence returned several times the same gene in several organisms. In such cases, we reported the version of the gene that gave the best hit (Figure 5E and F). One of those genes, Q96J66, reported on LG1, codes for an ATP binding cassette protein that has previously been described as multi-drug resistance associated protein [68]. In addition, the P-glycoprotein (Pgp), a member of the ATP-binding cassette (ABC) transporter protein superfamily was recently reported for playing an important role in EMB resistance [33, 69]. However, other studies could not establish significant correlation between Pgp polymorphisms and EMB susceptibility [70, 71]. A second candidate gene for drug resistance is Q28FI8 on linkage group 5, coding for Cytochrome b5 domain-containing protein. Cytochrome b5 is a known activator of cytochrome P450 [72], which has documented insecticide resistance effects in several species [73, 74]. This is concordant with the fact that EMB is one of the most extensively used chemical pesticides to control lice infestation in sea cages [25], and that intensive use of the substance led to development of resistance [27–30].

Because exposure to pesticides used for treating lice infestation is probably the strongest directional selection pressure applied to *L. salmonis* at the temporal scale of the present study, it is likely that the selective sweeps reported here are connected to positive selection on variants that provide better resistance to pesticides. The observations reported in the present paper would fit with this hypothesis: the region of the sweep on LG1 contains one candidate gene for a family of protein that has already been identified for playing a role in resistance to the most commonly used delousing chemical in salmon farms. Moreover, the use of the same chemical in all regions, as it is the case for in-feed lice infestation treatment, is expected to induce a strong selection pressure on the same resistance locus in all regions. This second fact also coincides with our observations of haplotypes under selective sweeps that are highly conserved across distant regions of the North Atlantic. Together, those facts point at pesticide resistance as the most likely cause for the observed selective sweeps.

One genomic region on LG14 displayed a number of SNPs with high F_ST_ values (Figure 2B), and was reported with significant signal for selective sweeps in Faeroe Island and Shetland samples (Additional file 4: Figure S1). Within the 19 genes predicted in this region, one coded for acetycholinesterase, which is the well-known target for organophosphates. Because it has been established that a variant type of acetylcholinesterase provides resistance to organophosphates in lice [20], it is possible that the high values of F_ST_ among sampling sites and the weak signal the of selective sweep reported on LG14 are due to an older event of selection for lice resistant to organophosphates. Organophosphates were the only chemical used to control sea lice up to around 1995 and major resistance problems occurred and left the chemical largely ineffective. However, due to emerging resistance against both EMB and phyretroids, Azametiphos (an organophosphate) was reintroduced and have been used alone and in combination with other medicines during the last 6–7 years.

### Link between EMB resistance and selective sweeps

The possible link between the detected selective sweeps and pesticide resistance was specifically investigated for EMB, which is the most commonly used pesticide to control *L. salmonis* in the Atlantic in the period 2000–2010. A genome-wide association analysis revealed that a broad region around the selective sweep of LG5 had a strong and significant contribution to the variation in EMB resistance (Figure 7). This last analysis demonstrates a clear link between genetic variation on LG5 around the selective sweep region, and EMB resistance. The selective sweep region on LG1 did however not show any link to EMB resistance. This could indicate that despite coinciding with a coding sequence for a protein that has previously been described as multi-drug resistance associated [68], the selective sweep on LG1 is caused by another factor than resistance to EMB. Alternatively, we point out that the lice that were included in the genome wide association dataset were all sampled in South Norway, whereas in the population genetic dataset only the samples from Ireland, Shetland and Faeroe Island displayed highly frequent haplotypes under the selective weep region on LG1. It is thus possible that the variant under positive selection on LG1 were not present in the association dataset.

We also point out that the power to detect association between genomic regions and EMB resistance was better in the F2 dataset than from the F1 cross between sensitive and resistant forms. In the F2 dataset, 5 genomic regions were significantly associated with the resistance while only two in the F1 data. When the same region was significantly associated in both datasets, the association score was larger in the F2 data.

An F2 intercross represents a notoriously better experimental design to maximize the genotypic variability in a population, while F1 crosses are not suitable for populations with fixed allelic frequencies. Assuming that the resistant and sensitive lines had fixed allelic frequencies at loci associated with EMB resistance, the resulting F1 hybrids would all be heterozygous at those loci, thus reducing to zero the genotypic variability of that population together with the power to associate genotypes to phenotypes.

## Conclusions

We produced the first SNP array for *L. salmonis*, and used it to genotype a set of geographically distinct samples in order to assess whether it was possible to identify signatures of pesticide resistance and dispersal, as well as to evaluate population genomic structure of this economically and ecologically important parasite. All results supported the hypothesis that this parasite is characterized by a single panmictic population through its distribution in the Atlantic. Importantly, the search for signatures of positive selection revealed two genomic regions under strong positive selection. Both of the selective sweeps identified coincided with the coding sequence of one or several possible candidate genes for pesticide resistance. Additionally, one genomic region on LG4 displayed a weak signal for selection, which may correspond to an older selective sweep caused by the development of resistance to organophosphates which have been used for a longer period. Genome-wide association analysis revealed a strong link between the selective sweep on linkage group 5 and resistance to EMB which was the most commonly used pesticide between years 2000 to 2010, when the samples upon which the study is based were collected. The strong selective sweeps identified on linkage groups 1 and 5 were both characterized by haplotypes that were present in high frequency in samples from distant regions of the North Atlantic. This last observation strongly suggests that *L. salmonis* has a high capacity to spread new advantageous mutations across continents in the time scale of just a few generations or years (at most 11 years), thus corroborating concerns that pesticide resistance can develop and rapidly spread over large areas on an ecological time-scale. These results have very significant implications for the way in which pesticide resistance for *L salmonis*, and potentially other parasitic copepods, is managed in marine aquaculture.

## Methods

### Generating sequences from *L. salmonis*

DNA sequences were obtained from *L. salmonis* samples collected in 2009 from five different regions of the north Atlantic. Four of these sets of samples have been described previously [39]; C858 (Canada), S856 (Shetland), I852 (Ireland), N849 (Northern Norway) (Figure 1). The fifth sample was collected in September 2008 from an emamectin-benzoate-desensitized population in Austevoll, Western Norway. Eight adult female *L. salmonis* were sampled from each of the five regions [75]. For all samples, DNA was isolated in a 96-well format using the DNeasy kit according to the manufacturer’s instructions (Qiagen, Hilden, Germany). Equal amounts of DNA from each of the eight individuals from each station were pooled to meet concentration demands and were sequenced by Fasteris SA using the Illumina HiSeq 2000 platform following their standard protocols [75]. In addition to the five regional samples, DNA was also sequenced from Expressed Sequence Tags (EST). In total, 715.10^6^ reads were generated from the five regions and EST samples, with an average length of 100 bp/read.

### SNP detection and chip design

The SNP selection process involved 715.10^6^ paired-end Illumina reads from the five geographical regions, and one set of 93673 single-end reads from EST sequences. Reads were first quality filtered and trimmed using the FASTX-Toolkit http://hannonlab.cshl.edu/fastx_toolkit/. Fastq quality trimmer was used to discard all nucleotides with quality score lower than 28. Fastx clipper was used to remove adapter sequences matching the following ten-bases sequences: GAAGAGCGGT. Reads were also filtered for repeat sequences using repeat-masker (http://www.repeatmasker.org). Each filtered paired-end read was aligned to the most recent genome assembly available (http://sealouse.imr.no/), using the BWA aligner [76]. The six regions’ alignments were individually checked for polymorphism using the samtools pileup function [77]. SNPs to be included in the 6 k array were divided into four groups:

1. The largest fraction of the SNPs were selected to provide a regular coverage over the 700 Mb genome assembly. To optimize the genome coverage, SNPs were chosen with intervals of 100 Kb. SNPs were thus chosen from the largest scaffolds available, with 100 Kb bases interval on scaffolds larger that 100 Kb, or only one SNP per scaffold, for scaffolds smaller than 100 Kb. SNPs also had to match the following quality criterion: having a minimum of two reads depth per region in at least four regions out of five, and to be polymorphic (displaying both reference and alternative nucleotide) in at least three regions.
2. A second set of 186 SNPs was selected in genomic regions aligned with EST sequences. These SNPs had a minimum of two reads depth in at least three regions out of five.
3. A third set of SNPs was selected to help discriminate individuals from the five geographical regions, based on genotype information. In this group, SNPs were selected for having coverage deeper than two reads in at least three regions. There are ten pair-wise combinations of the five geographical regions. For each pair, a set of SNPs was chosen for being monomorphic for one allele in the first region, monomorphic for the alternative allele in the second region, and polymorphic for the remaining region(s), providing thus a possible tool for determining the origin of individuals, based on genotype information. Fifty of such diagnostic SNPs were selected for each regional pair, with the exception of two pairs: Ireland-Shetland and Ireland-Canada where only 35 diagnostic SNPs could be found for each pair. Based on the same principle of selection, fifty SNPs were selected for discriminating West Atlantic (Canada) from the East Atlantic (Ireland Shetland Austevoll Norland) regions, and fifty SNPs were also selected for discriminating Austevoll (showing resistance to chemical treatment) from the other regions.
4. Finally, a set of 174 SNPs was selected at narrow intervals within the same scaffolds in order to provide information about linkage disequilibrium (LD) in salmon lice populations. These SNPs had a minimum of two reads depth in at least two regions out of five, and were polymorphic in at east one region. These 174 “LD” markers were distributed on 24 scaffolds among 11 linkage groups, and separated by an average of 10Kb when situated on the same scaffold.

### DNA samples

A total of 576 samples were collected from twelve sampling sites in six regions in the North Atlantic (Figure 1). Five of the sampling sites were the same as those used to produce the DNA libraries previous to SNP detection: C858 (Canada), S856 (Shetland), I852 (Ireland), N849 (Northern Norway), and N813 (South Norway) [39]. In addition, genotypes were also obtained from seven sites also described in previous reports [39]; N854 (South Norway), N837 (North Norway), F850 and F851 (Faeroe Islands), S855 (Shetland), I853 (Ireland), C857 (Canada), (Figure 1). The samples are thus divided in six geographical regions and two sampling sites per region. A total of 48 individuals were genotyped in each sampling site.

All 576 samples were quantified using picogreen fluorescent stain (Invitrogen, USA) and an aliquot from each examined on 1% agarose gel to subjectively assess DNA quality based on the presence of high molecular weight DNA. Where necessary, samples were concentrated using a Speed-vac to fall within the target concentration range of 25-75 ng/ul required for genotyping. Genotyping was performed according to the infinium HD assay protocol (Illumina, San Diego). SNP genotyping results were quality checked to eliminate unreliable markers. Clusters were inspected using the Genotyping Module within Genome Studio. Genotype data from 5090 polymorphic SNPs were obtained for the 576 samples.

### Outlier detection

Markers were tested for Hardy-Weinberg equilibrium in each population. Markers with allele frequencies that deviated significantly (p <0.01 with Bonferroni correction) from HW equilibrium in at least one of the populations were considered outliers. Scans for loci under selection were conducted with three different software: *i)* The F_ST_ outlier based approach Fdist [78] implemented in LOSITAN [79], *ii)* The Bayesian approach implemented in BayeScan [80], and *iii)* the hierarchical F statistic F_CT_ approach [81] implemented in Arlequin 3.5 [82]. Both approaches use the F_ST_ measurement of genetic distance as basis for detecting directional selection. In *i)* and *iii)*, directional selection is revealed by the distribution of the ratio of the genetic distance by heterozygicity (F_ST_/He), whereas in *ii)*, the genetic distance among populations is decomposed in two components: a population-specific component (Beta) shared by all loci and a locus-specific component (Alfa). In the later, directional selection is revealed by a significant value of Alfa. In *iii)* the data is stratified by population in a hierarchical manner, from local demes to the largest population. The three approaches handle the detection of markers under selection in a different manner and are expected to produce slightly different results.

Two alternative datasets were generated: one dataset (noted 6P) where the two sampling sites within each region are assumed to come from the same population, and one dataset (noted 12P) where each sampling site is considered as a different population. In total, five genome scans were performed: Both 6P and 12P datasets were scanned with LOSITAN and BayeScan, while the F_CT_ approach was only used on the 12P dataset because it already takes into account the hierarchical structure of the data and uncertainty regarding the number of demes in the population structure [81, 82]. After detection of outlier markers, the data were split into two sets of markers: markers under selection if reported as outlier by three or more of the above-mentioned genome scans, and neutral markers if reported as neutral by both genome scans. This criterion produced one set of 4810 neutral markers and 167 markers under selection.

### Genomic regions affected by directional selection

In addition to scan for individual outlier marker, the genome was scanned for regions of reduced genetic variation linked to a recently fixed beneficial mutation, or selective sweeps. At the molecular level, selective sweeps are characterized by a reduction of the genetic variability in the flanking sequences from both sides of the selected locus [44] and a local increment of linkage disequilibrium, also from both sides of the selected locus [64], Based on these two features, two summary statistics were used to assess the presence of selective sweeps. The first approach is a composite likelihood ratio (CLR) based on site frequency spectrum [44, 83], and implemented in SweeD software [84]. This method consists in scanning the genome for regions of reduced genetic diversity. The second approach is the Omega statistic [64] implemented in the OmegaPlus software [85], which scans the genome for local patterns of high linkage disequilibrium. Both methods were applied to scan the 5090 SNPs genotypes from 576 individuals. For genomic regions reporting significant evidence of selective sweeps, haplotypes were reconstructed independently within each sampling site with Phase2.1 software [86, 87].

The significance thresholds of the scans for selective sweeps were obtained by bootstrapping after simulating 1000 data sets under neutral model. Simulations were performed with MSMS software [88].

SweeD detects deviations in the site frequency spectrum from neutral expectations, however, here, we work with a set of SNP that were selected based on allelic frequencies from a small subset of individuals. This is expected to shift the frequency spectra even in the case of all markers being neutral. To include this potential bias in the computation of significance threshold, the null dataset was simulated to reflect the SNP selection process as follow:

1. 600,000 polymorphic sites were simulated on 576 individuals.
2. The 576 individuals were divided in 12 groups of 48 individual each, as in the sampling sites of the present data.
3. Eight individuals were randomly picked from five groups in order to replicate the pooled DNA sequences that were used for the SNP selection process.
4. From the sites that matched the SNP selection criteria (being polymorphic in at least three groups out of five), 6000 were randomly picked to generate a neutral dataset of 6000 SNP genotypes on 576 individuals.

### Gene prediction

SNPs determined to be under selection were mapped to preliminary genome scaffolds and compared to a draft gene annotation (http://sealouse.imr.no/). The identified scaffolds had an average length of 440 Kb, and possible genes present on these scaffolds were predicted by *i*- *ab* initio gene prediction [89, 90] on the scaffold sequence, and *ii*- functional annotation of genes based on BLAST hits [67]. The predicted peptide sequences were compared to SwissProt using BLASTP with E-value threshold of 10^-6^.

### Genome-wide association mapping for drug resistance

In order to investigate the possible link between selective sweeps and pesticide resistance, a second set of lice were also genotyped on the SNP array. This second dataset consisted of 576 individuals divided in 12 full sibling families that originated from three *L. salmonis* source strains with different tolerances of EMB [31]. Strains had been maintained in the salmon louse rearing facility using protocols previously described [91]. Five families were produced as hybrids between two laboratory strains, expected to be ‘tolerant’ and ‘susceptible’ to EMB [30], while the remaining seven full sibling families were produced as the F2 generation of the third source strain, that had uncertain level of tolerance to EMB. The resistant strain was collected in September 2008 near site N813 (Figure 1) after reports of Slice® treatment failures, while the susceptible strain was collected in October 2009 in Oslofjorden, east of Norway where no commercial salmon farming is taking place and exposure to EMB is low. The third strain was collected in April 2010 near site N813 (Figure 1), in a salmon farm where no reduced efficiency of Slice® had been reported despite being in a dense farming area.

The offspring of each family were exposed to EMB according to the following protocol: all individuals were placed in 5 L glass beakers and exposed to a one-dose (50 ppb) EMB overnight trial, performed by the method for bioassay testing described in Handbook in resistance management [92]. After 20 hours of exposure, each individual was evaluated in accordance with the response criteria, defined as ‘Living’ or ‘Moribund and/or dead’, and stored on 95% ethanol for subsequent DNA analysis [31]. All individuals were genotyped on the SNP array, and the software FastPhase [93] was used to reconstruct haplotypes in all linkage groups. SNPs were then pooled in groups of three flanking markers haplotypes, in sliding windows along the genome. At each genomic position, a hierarchical generalized mixed model with binomial family was fitted with the R package hglm [45]. The model included the status after exposure (dead or alive) as binary response to one fixed full sib family effect and one random haplotype effect. The dispersion coefficient of the random haplotype effect was kept as indicator of the genetic contribution to the variance in survival after EMB exposure.

The genome scan was repeated 1000 times with a randomized response vector to obtain an empirical distribution of the random genetic effect under null hypothesis. The upper 95 percentile of this empirical distribution was kept as 5% genome-wide significance threshold.

### Population structure

The genetic distance between samples was measured for each SNP with an R implementation of Weir and Cockerham formula [46, 94]. In order to test for possible isolation by distance among regions, pair wise genetic distance and geographical distance were compared with standard Mantel test [95] implemented in the R package vegan [94, 96]. Statistical significance of Mantel test was corrected a posteriori for multiple testing by applying Bonferroni correction. To investigate for possible population structure on the North Atlantic, the genotype data of the 4810 neutral SNPs was analyzed in the population genetic software STRUCTURE 2.3 [41] without population information, using 50,000 burnin, 200,000 iterations. The number of cluster (K) was tested for K = 1 to K = 12, with ten replicates for each value of K. Due to the long computation it requires, this analysis was performed on an 8-core workstation using the R package ParallelStructure [97] to distribute parallel runs of STRUCTURE on multiple cores. The number of cluster in the populations, K was then assessed by Evanno’s method [98] using the STRUCTURE HARVESTER implementation [99]. In addition, the genotype data was also analyzed with the “find.clusters” function from the R package ADEgenet [42, 94], also in order to infer the number of populations in the data.

### Population demography

Linkage disequilibrium was tested for each pair of markers by calculating the coefficient of correlation between genotype pairs r^2^ [100]. In addition, the effective size of each population (Ne) was estimated from population linkage disequilibrium with the LDNe software [48]. This program was implemented to estimate Ne from unlinked markers; therefore, we use a subset of 100 unlinked SNPs from the total set of 5090 (Additional file 5).

## Availability of supporting data

The data sets supporting the results of this article are included within the article and its additional files.

## Electronic supplementary material

Additional file 1: **List of 5091 SNP genotypes on 576 individuals.** The file contains 577 lines that correspond to the list of marker names (line 1), followed 576 lines corresponding to the 576 individuals genotypes. The first column lists the individual IDs with sampling site coded as in method section. (852 for Ireland, 849 for North Norway etc.…). The following 10182 columns correspond to the genotypes at each marker (two column per marker), with genotypes coded as either “1” “3” or “0”, where “0” corresponds to missing genotype. (ZIP 1 MB)

Additional file 2: Table S1: List of predicted genes on each scaffold under the selective sweep regions. (CSV 15 KB)

Additional file 3: Table S2: List of 6000 SNP selected to be included in the array together with flanking sequence region. (CSV 1 MB)

Additional file 4: Figure S1: Genome scans for selective sweep in all sampling sites with CLR and Omega statistics. (PDF 574 KB)

Additional file 5: **List of 100 SNP used for effective population size estimation.** (CSV 2 KB)
