# Supplementary material for: Human-induced evolution caught in action: SNP-array reveals rapid amphi-atlantic spread of pesticide resistance in the salmon ecotoparasite Lepeophtheirus salmonis
Source: BMC Genomics. 2014 Oct 26;15(1):937. doi: 10.1186/1471-2164-15-937 (PMC4223847; doi:10.1186/1471-2164-15-937)
Supplement: Supplementary file 4 — Additional file 4: Figure S1: Genome scans for selective sweep in all sampling sites with CLR and Omega statistics. (PDF 574 KB) [file 12864_2014_6634_MOESM4_ESM.pdf]

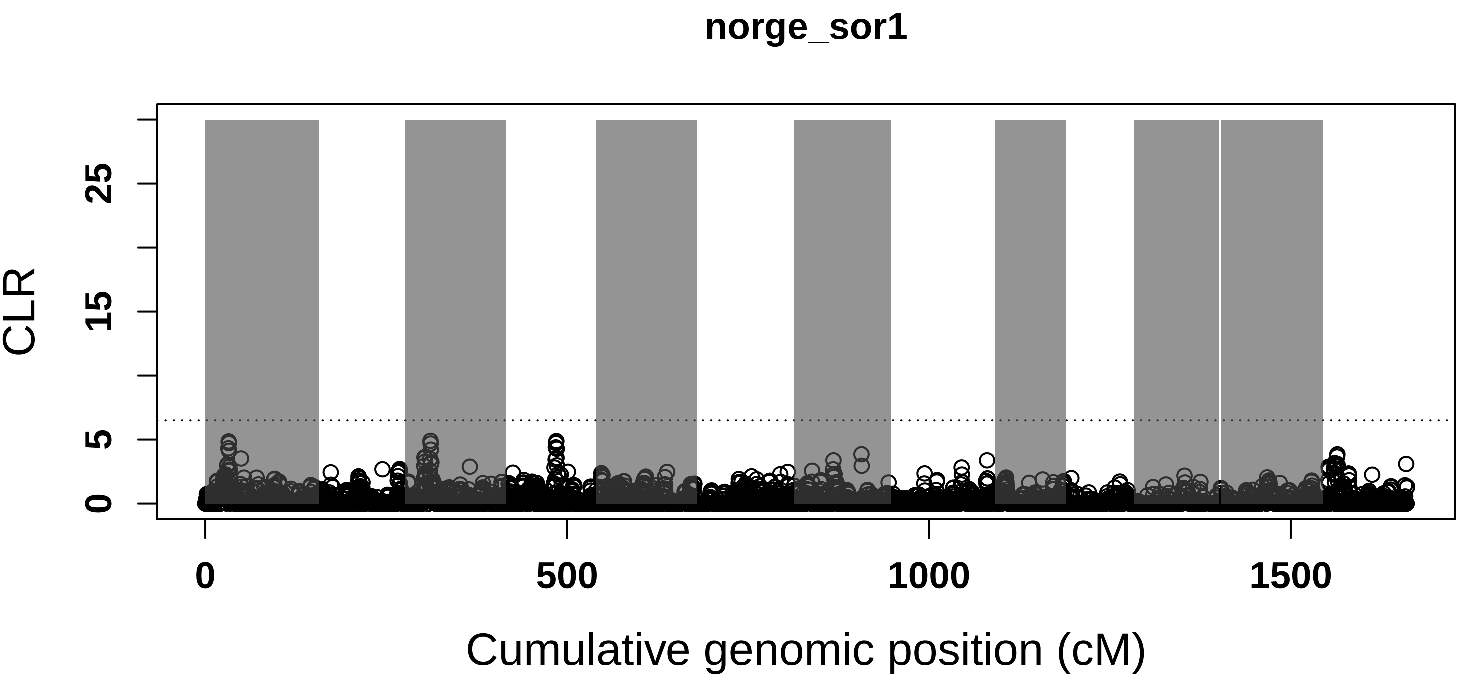
norge_sor1
CLR
0
5
15
25
0
500
1000
1500
Cumulative genomic position (cM)

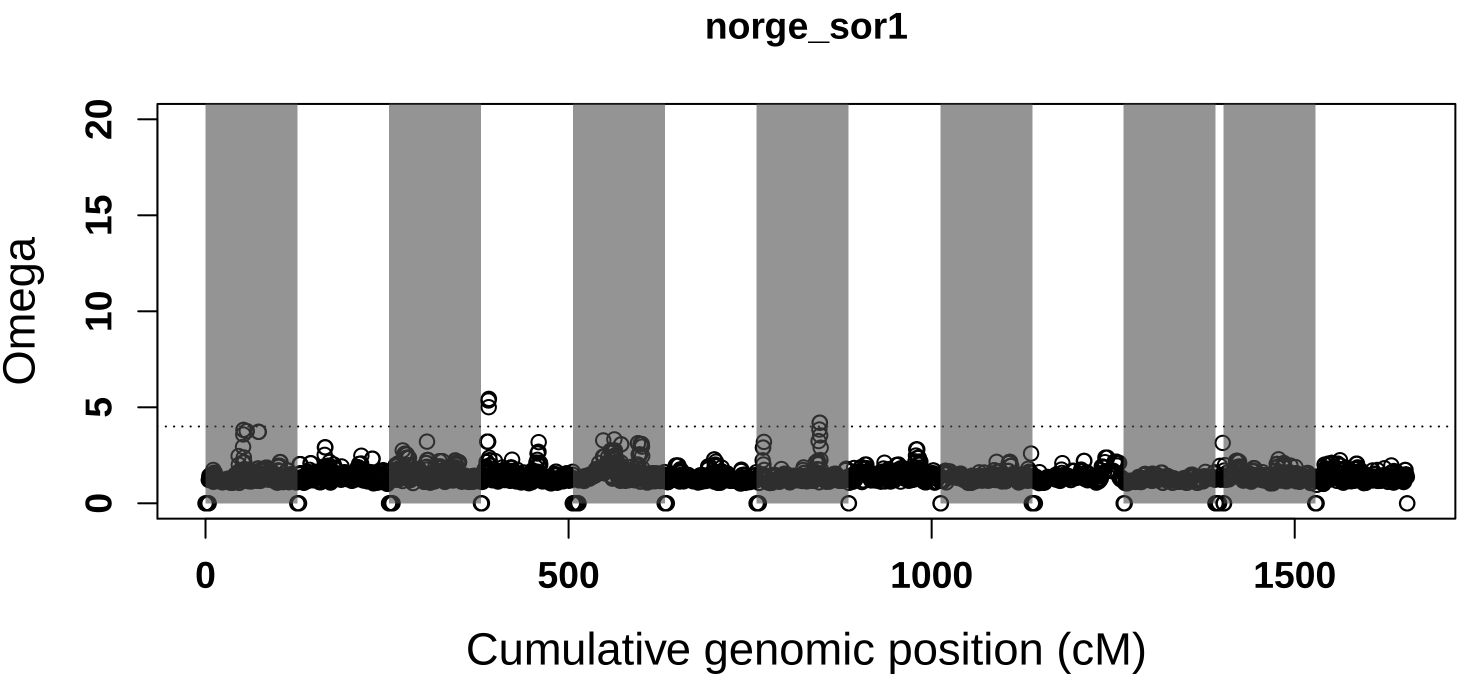

norge_sor1
Omega
0
5
10
15
20
0
500
1000
1500
Cumulative genomic position (cM)

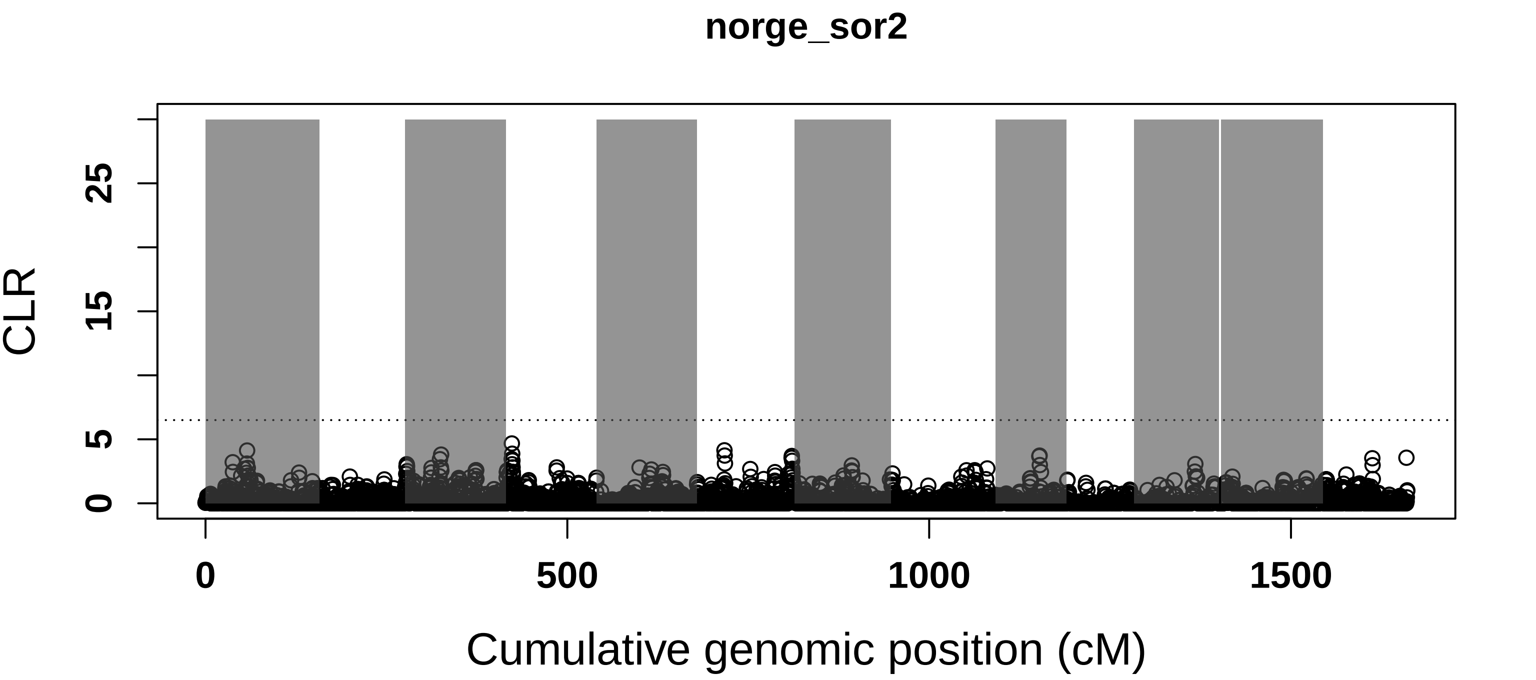

norge_sor2
CLR
0
5
15
25
0
500
1000
1500
Cumulative genomic position (cM)

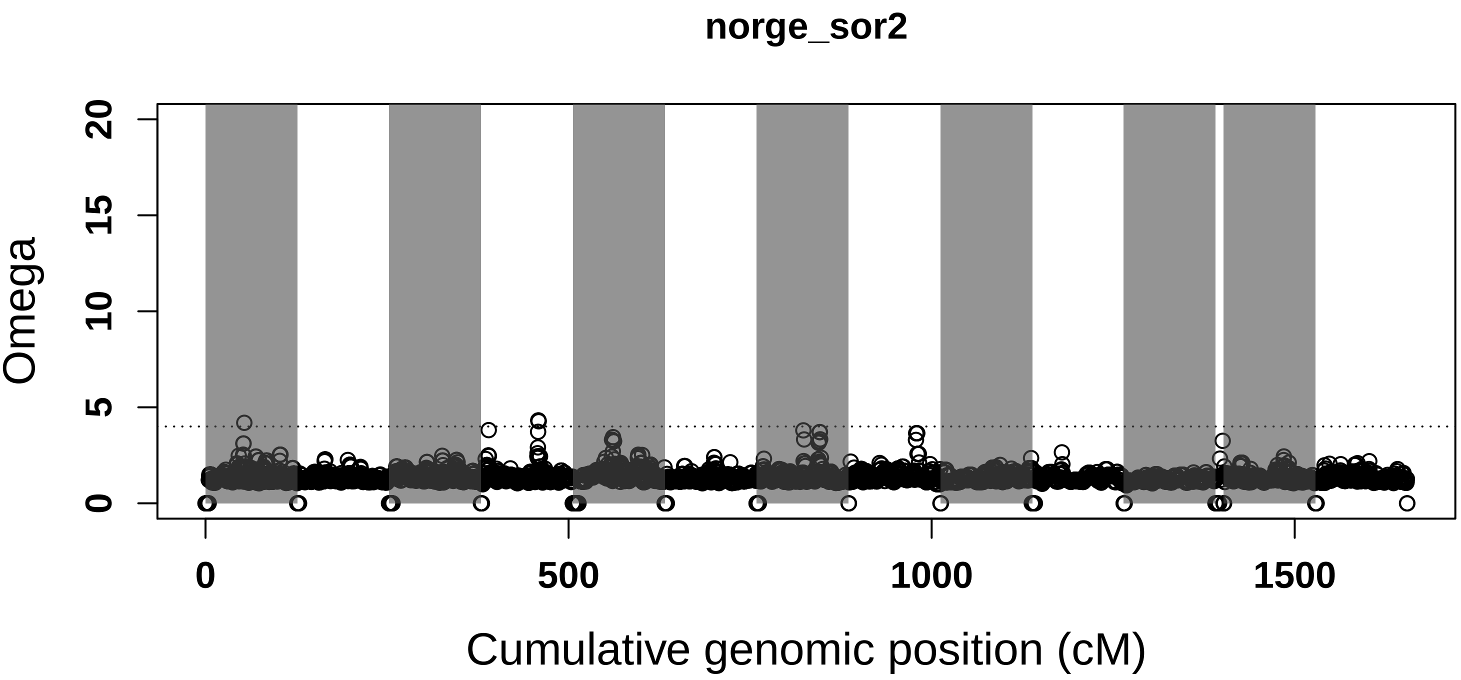
norge_sor2
Omega
0
5
10
15
20
0
500
1000
1500
Cumulative genomic position (cM)

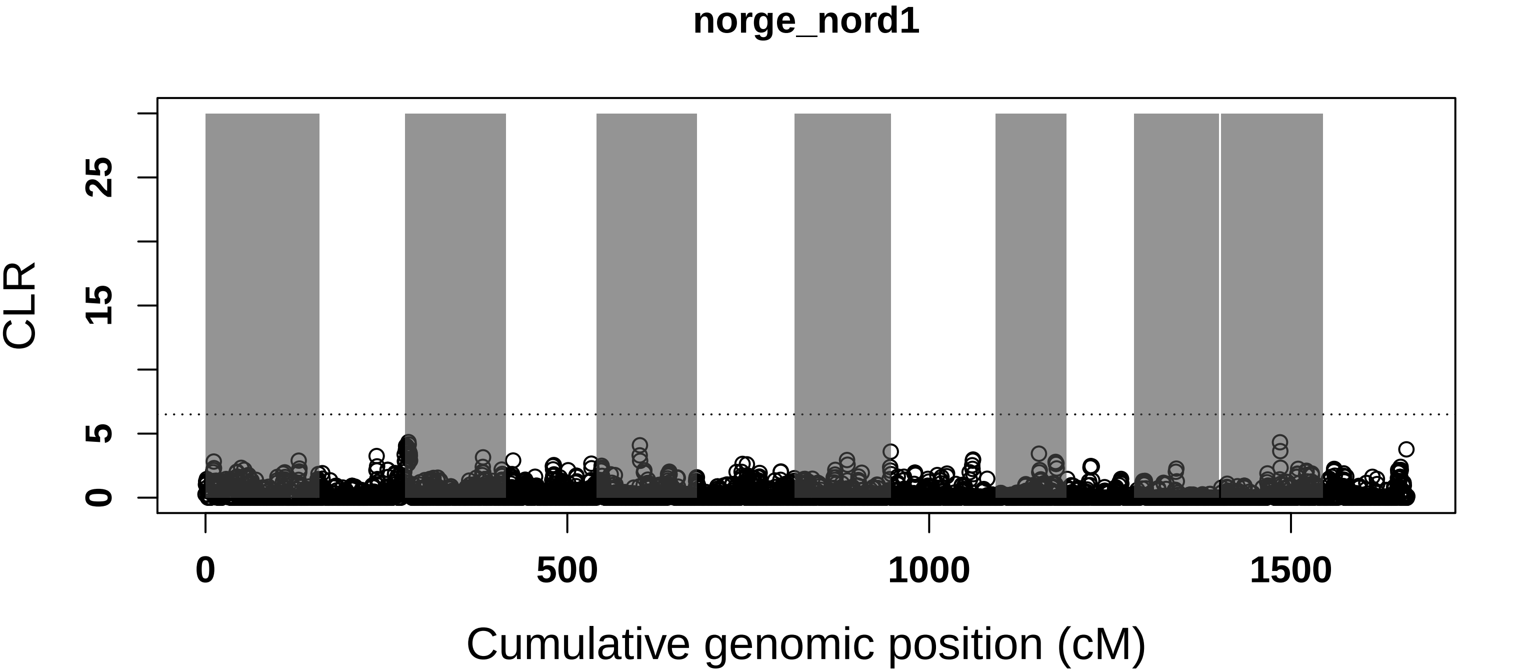
norge_nord1
CLR
0
5
15
25
0
500
1000
1500
Cumulative genomic position (cM)

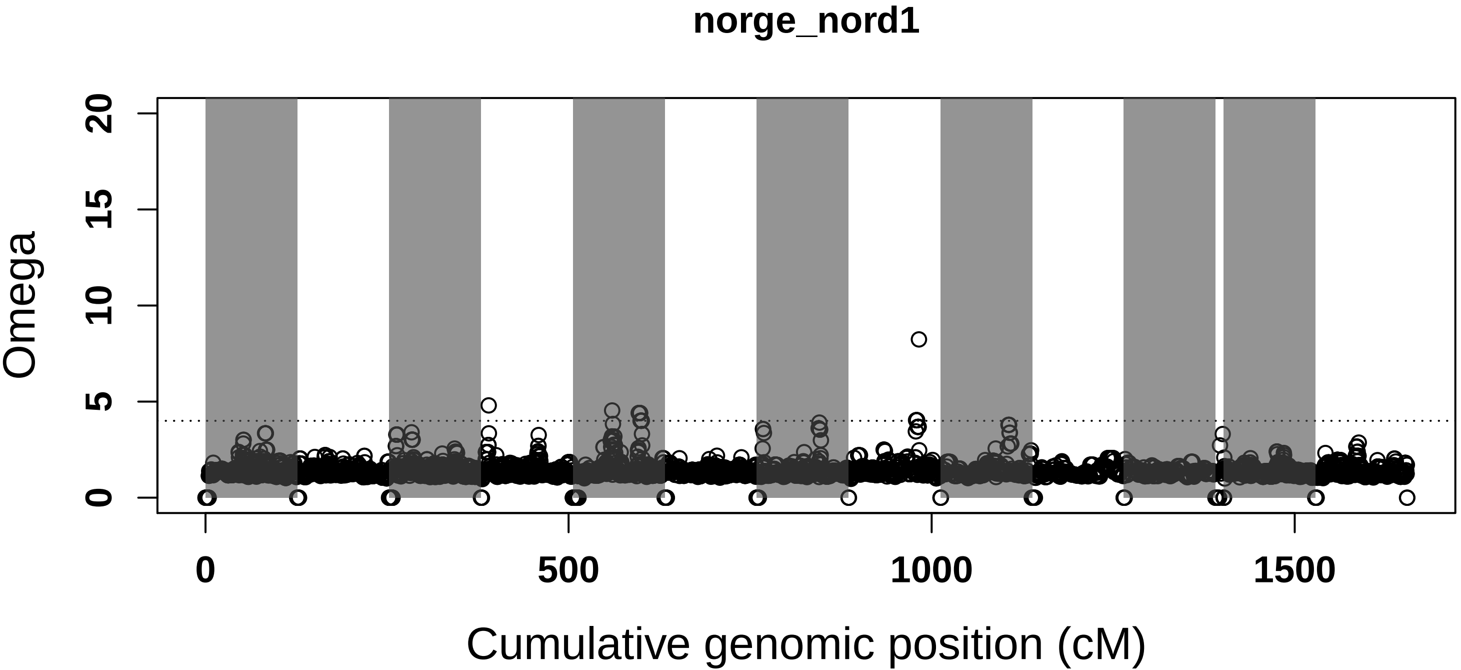

norge_nord1
Omega
0
5
10
15
20
0
500
1000
1500
Cumulative genomic position (cM)

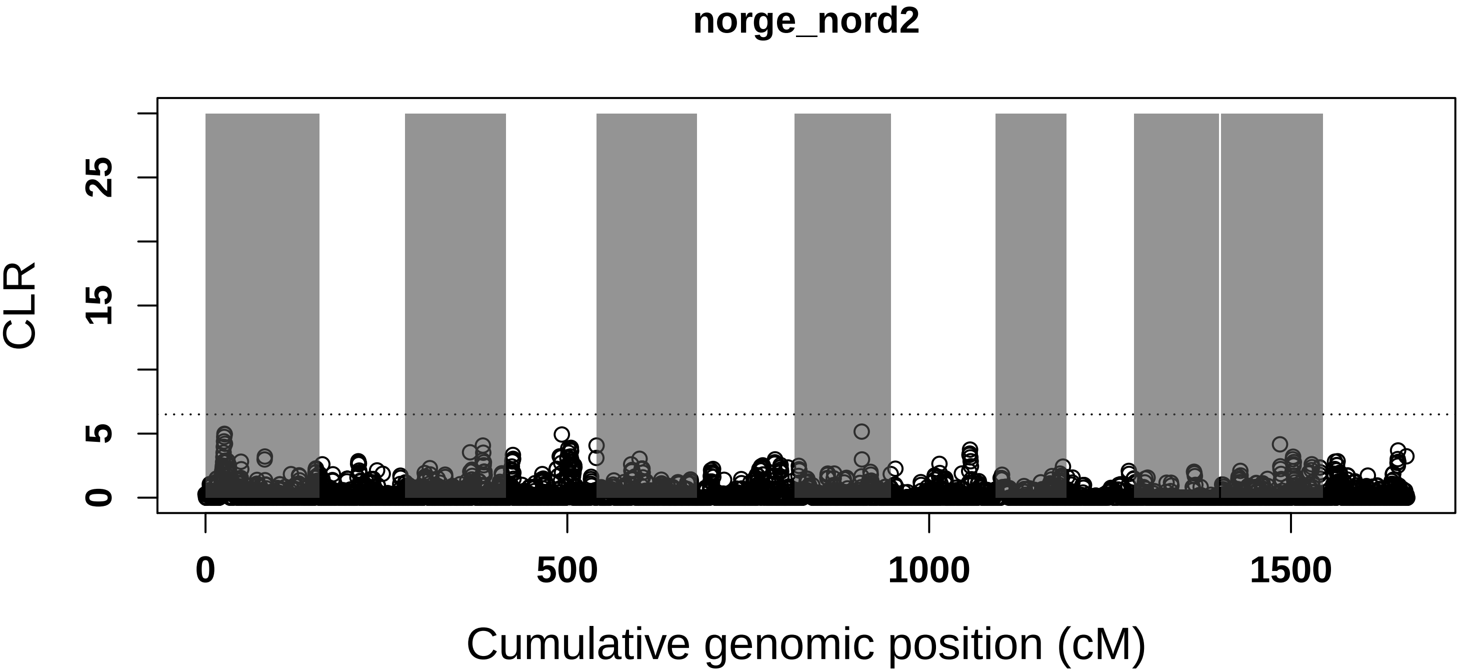

norge_nord2
CLR
0
5
15
25
0
500
1000
1500
Cumulative genomic position (cM)

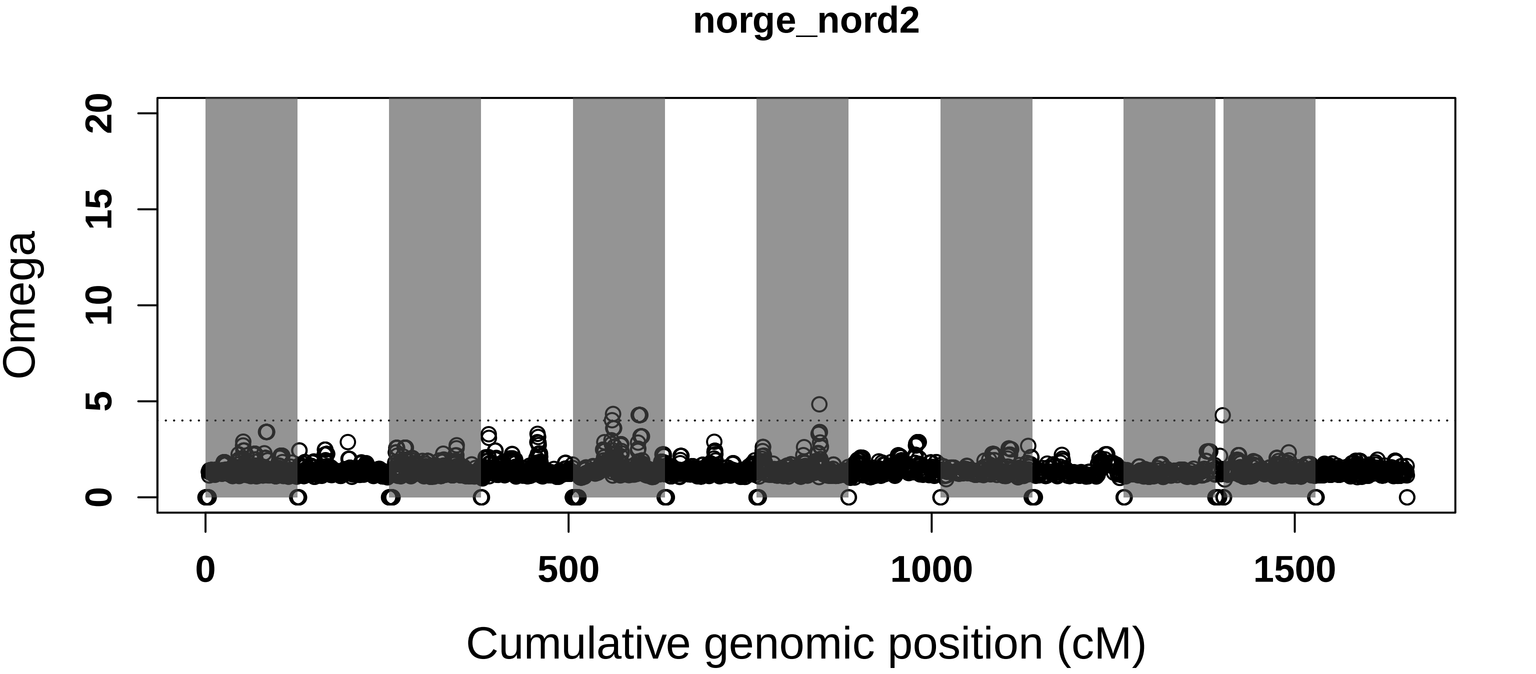

norge_nord2
Omega
0
5
10
15
20
0
500
1000
1500
Cumulative genomic position (cM)

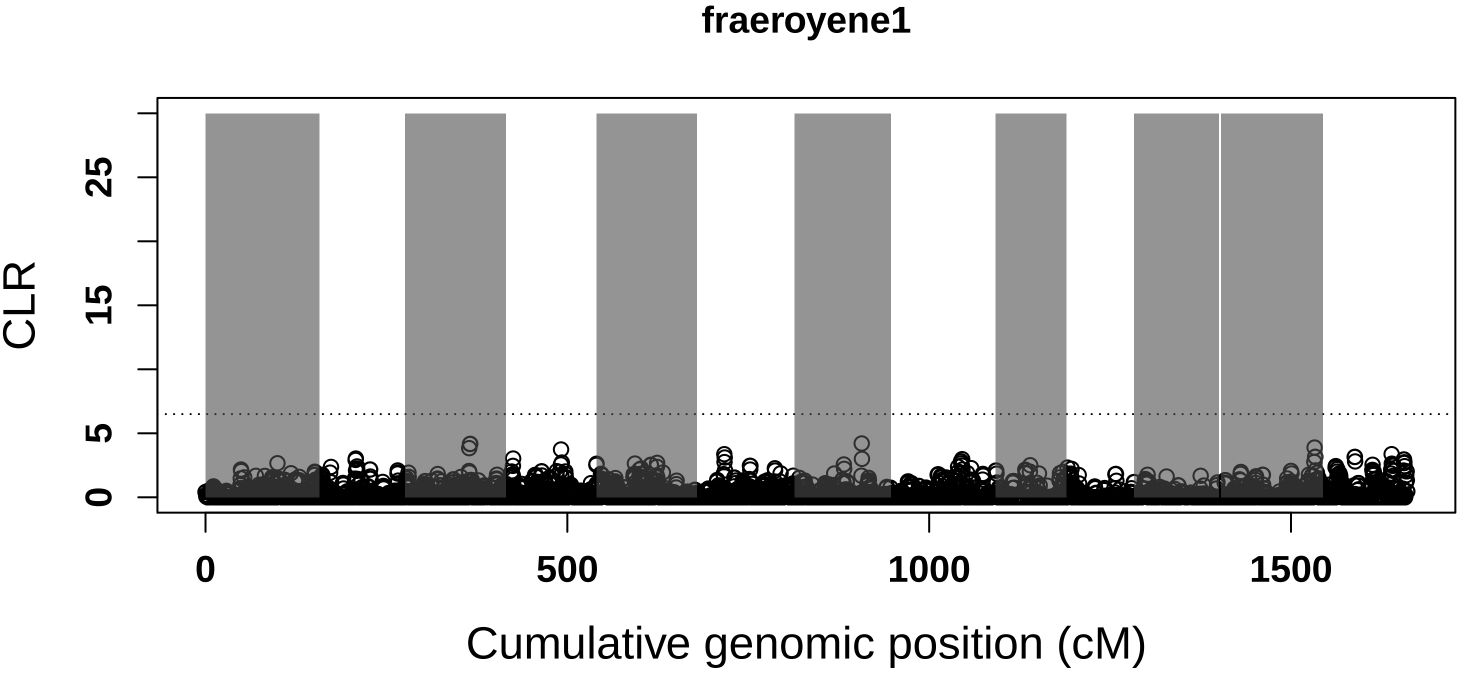

fraeroyene1
CLR
0
5
15
25
0
500
1000
1500
Cumulative genomic position (cM)

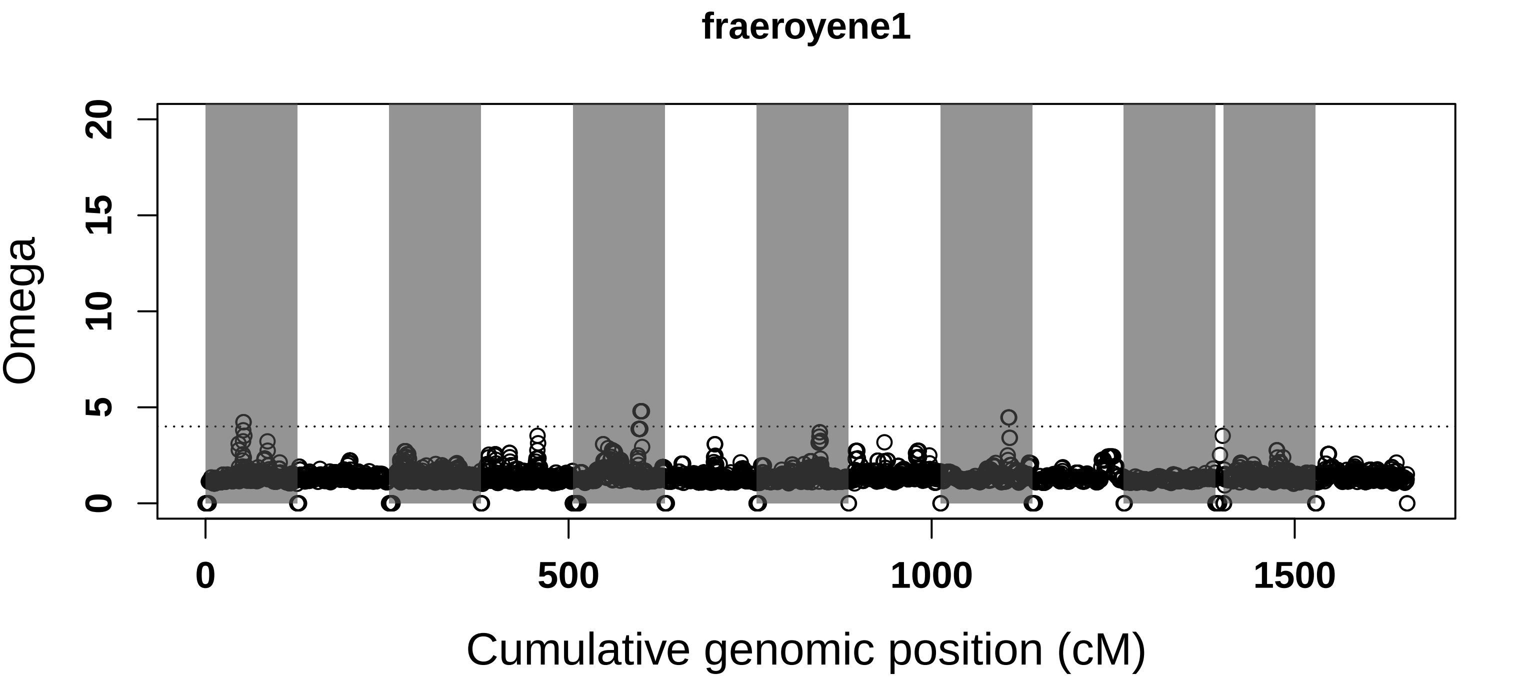
fraeroyene1
Omega
0
5
10
15
20
0
500
1000
1500
Cumulative genomic position (cM)

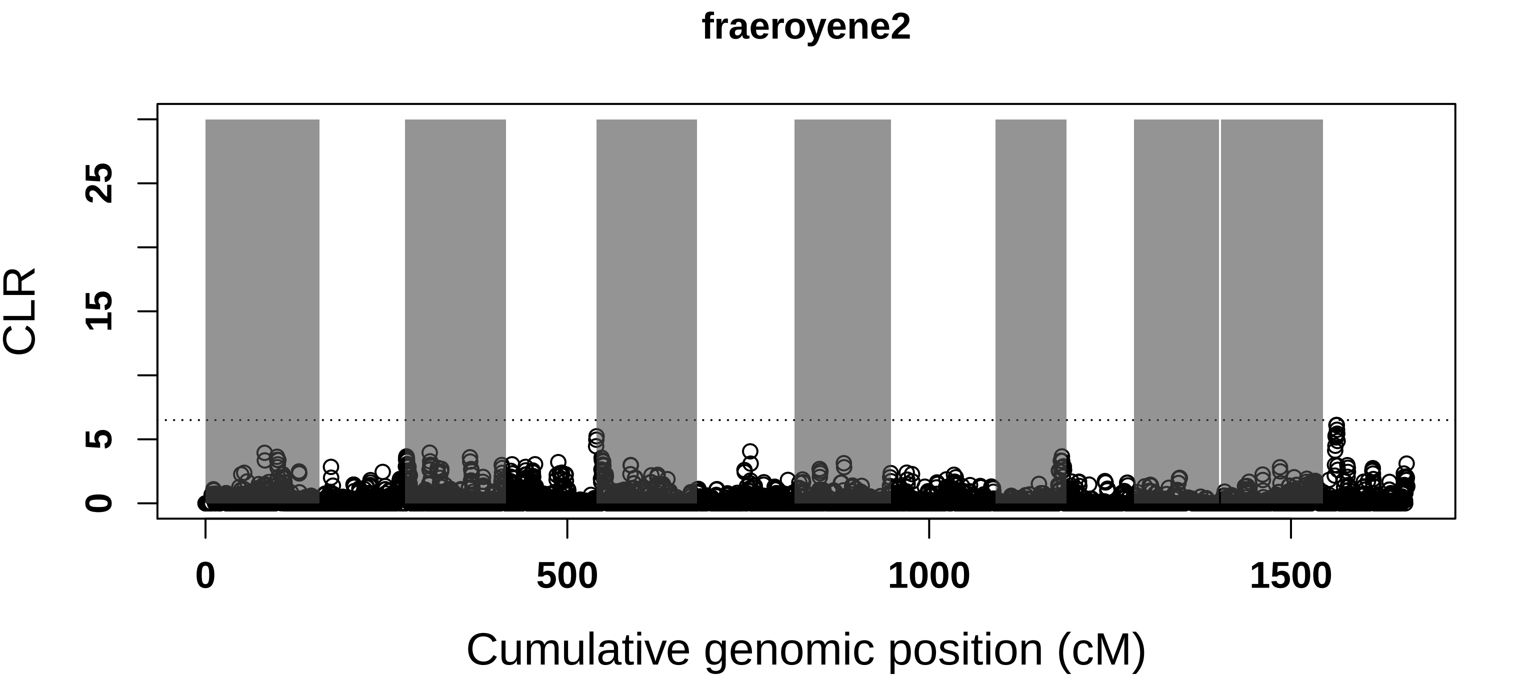

fraeroyene2
CLR
0
5
15
25
0
500
1000
1500
Cumulative genomic position (cM)

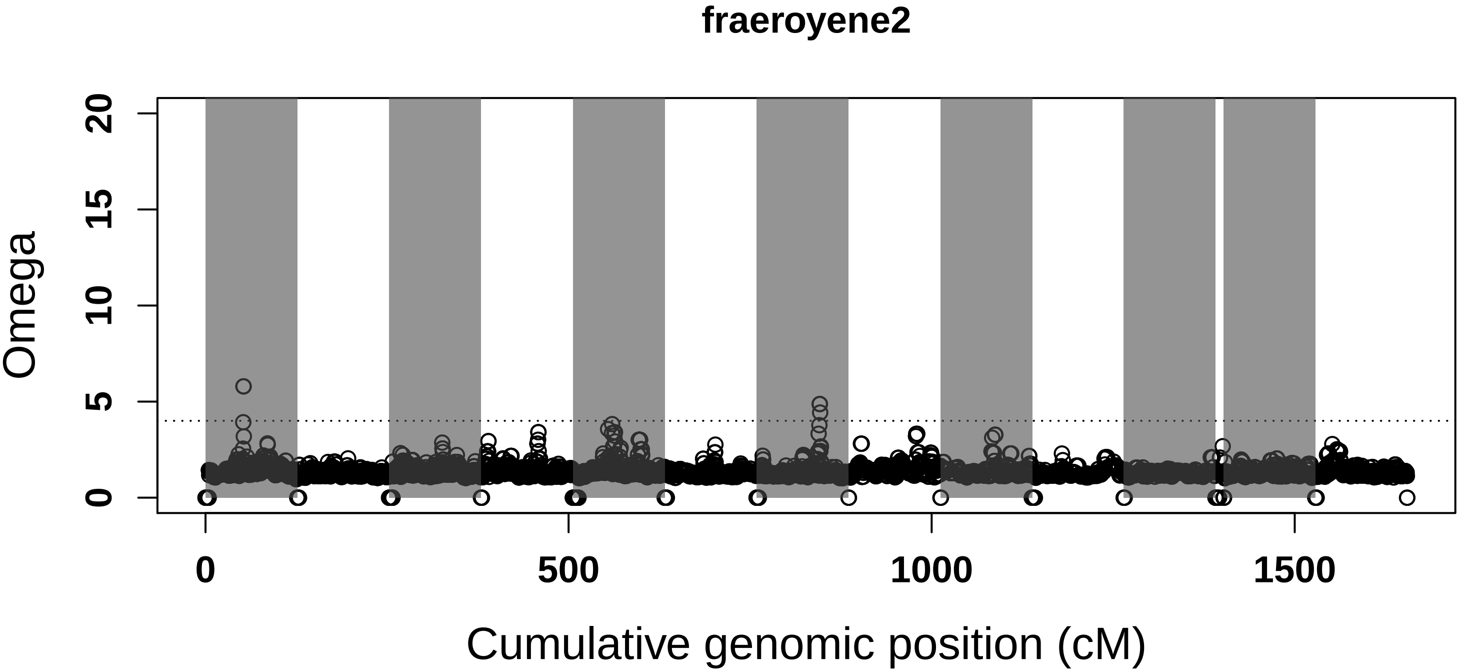

fraeroyene2
Omega
20
15
10
5
0
0
500
1000
1500
Cumulative genomic position (cM)

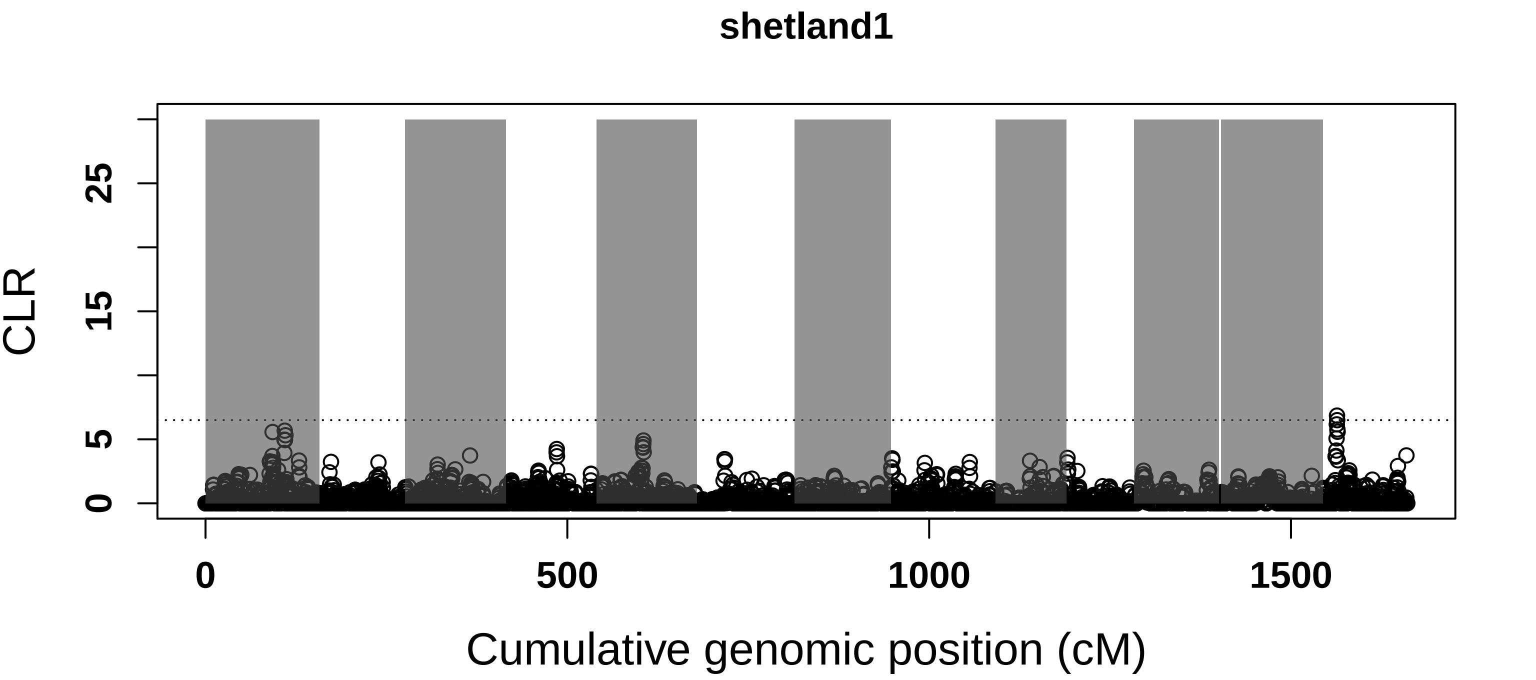

shetland1
CLR
0
5
15
25
0
500
1000
1500
Cumulative genomic position (cM)

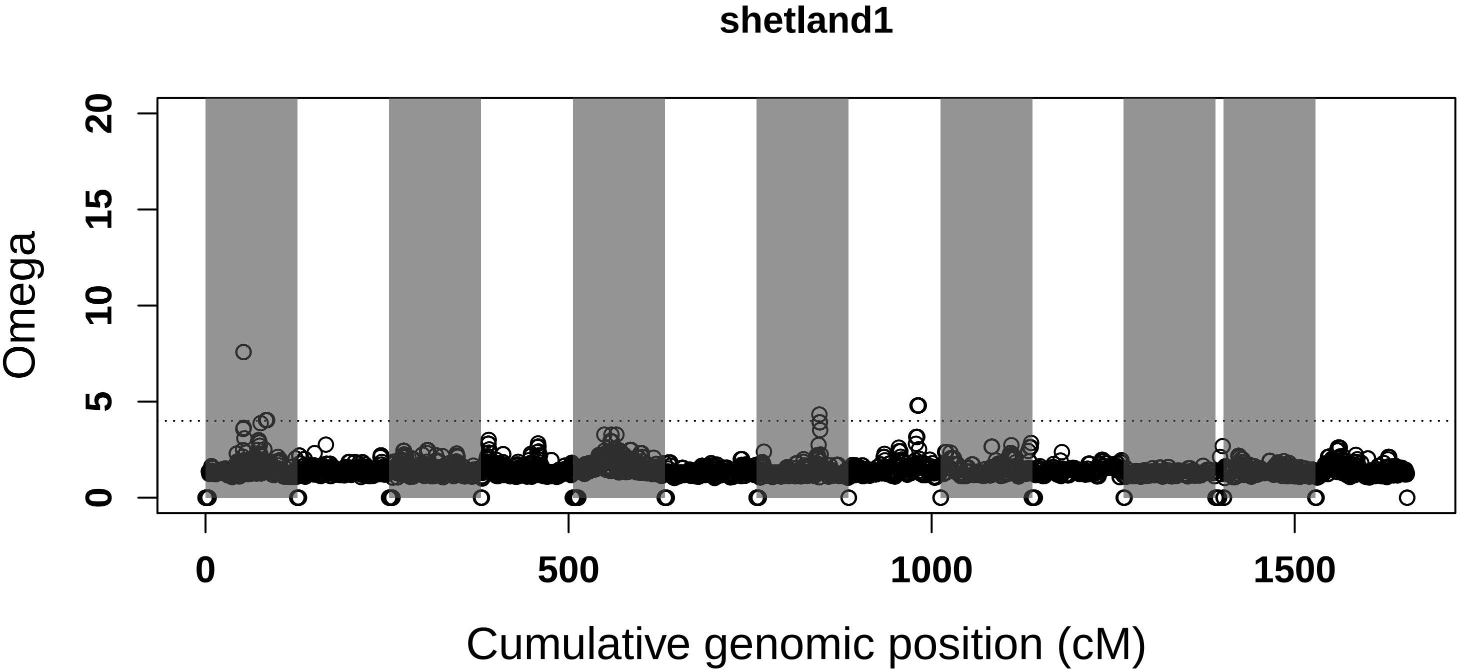

shetland1
Omega
0
5
10
15
20
0
500
1000
1500
Cumulative genomic position (cM)

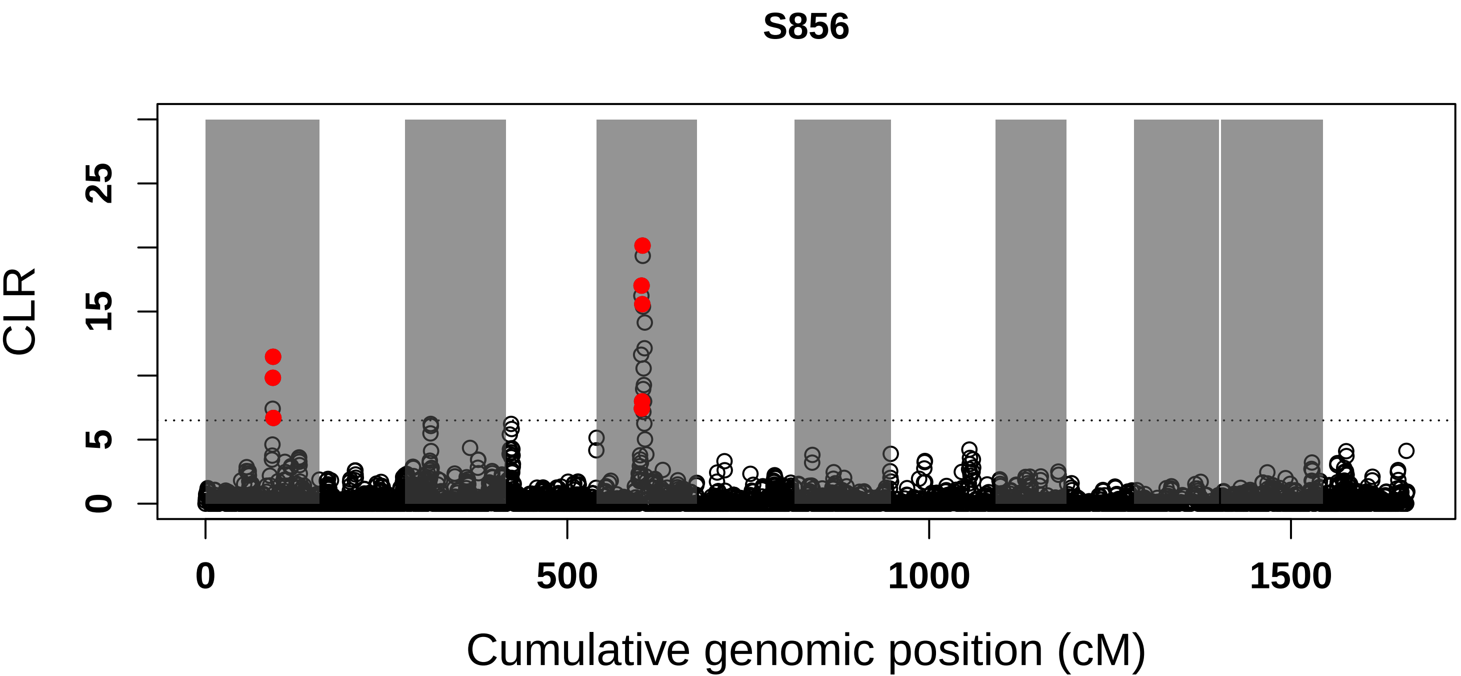
S856
CLR
0
5
15
25
0
500
1000
1500
Cumulative genomic position (cM)

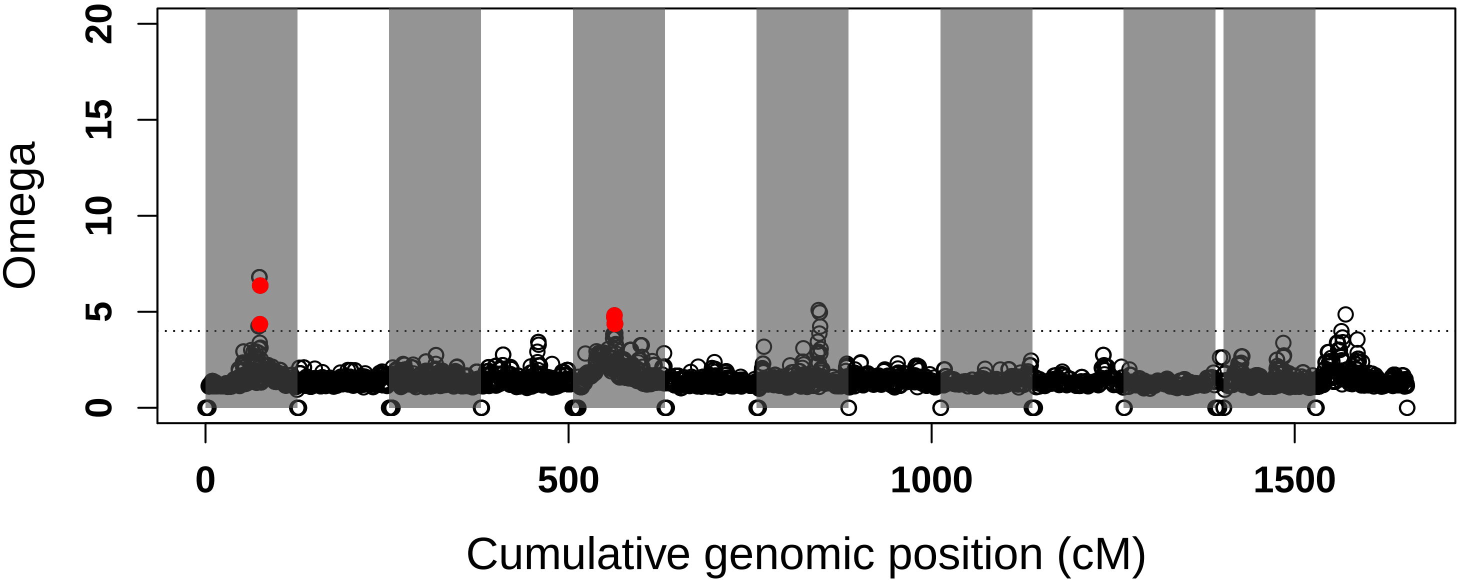

Omega
0
5
10
15
20
0
500
1000
1500
Cumulative genomic position (cM)

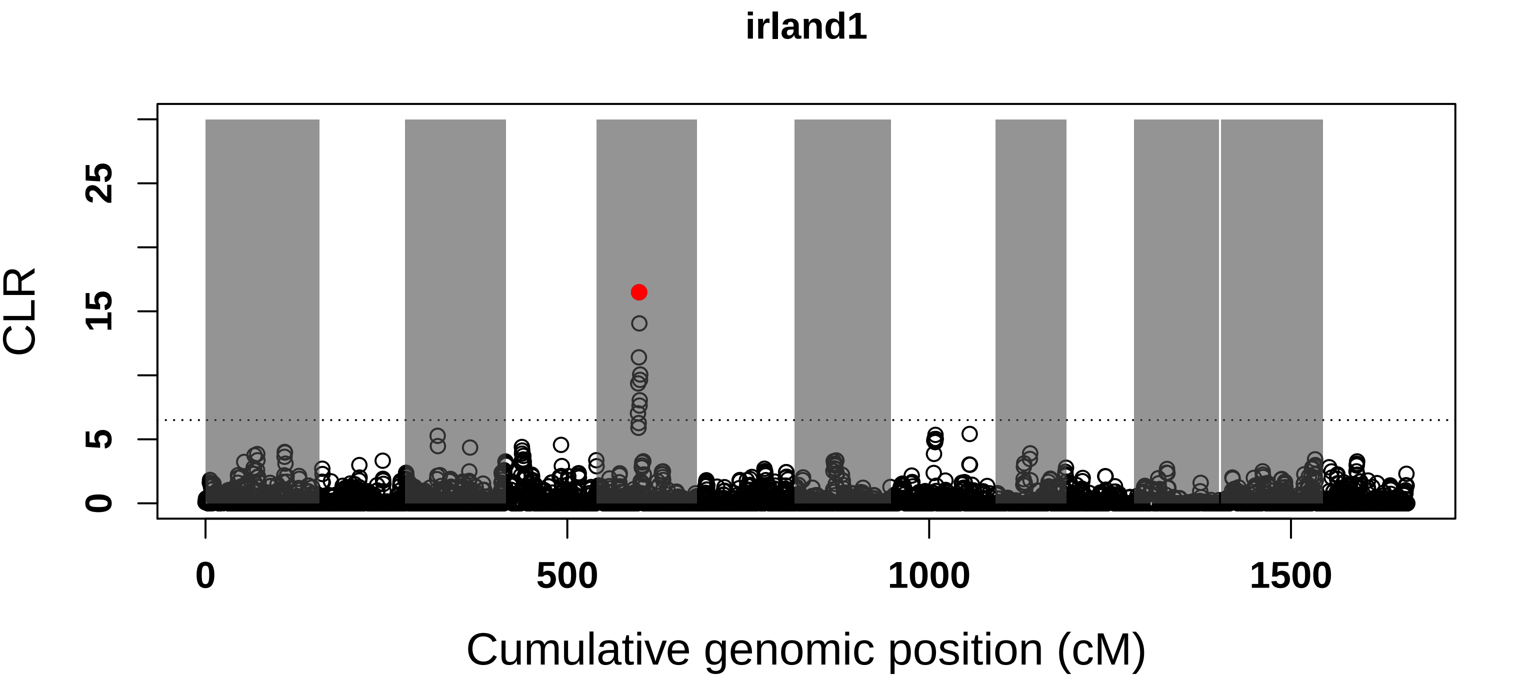

irland1
CLR
0
5
15
25
0
500
1000
1500
Cumulative genomic position (cM)

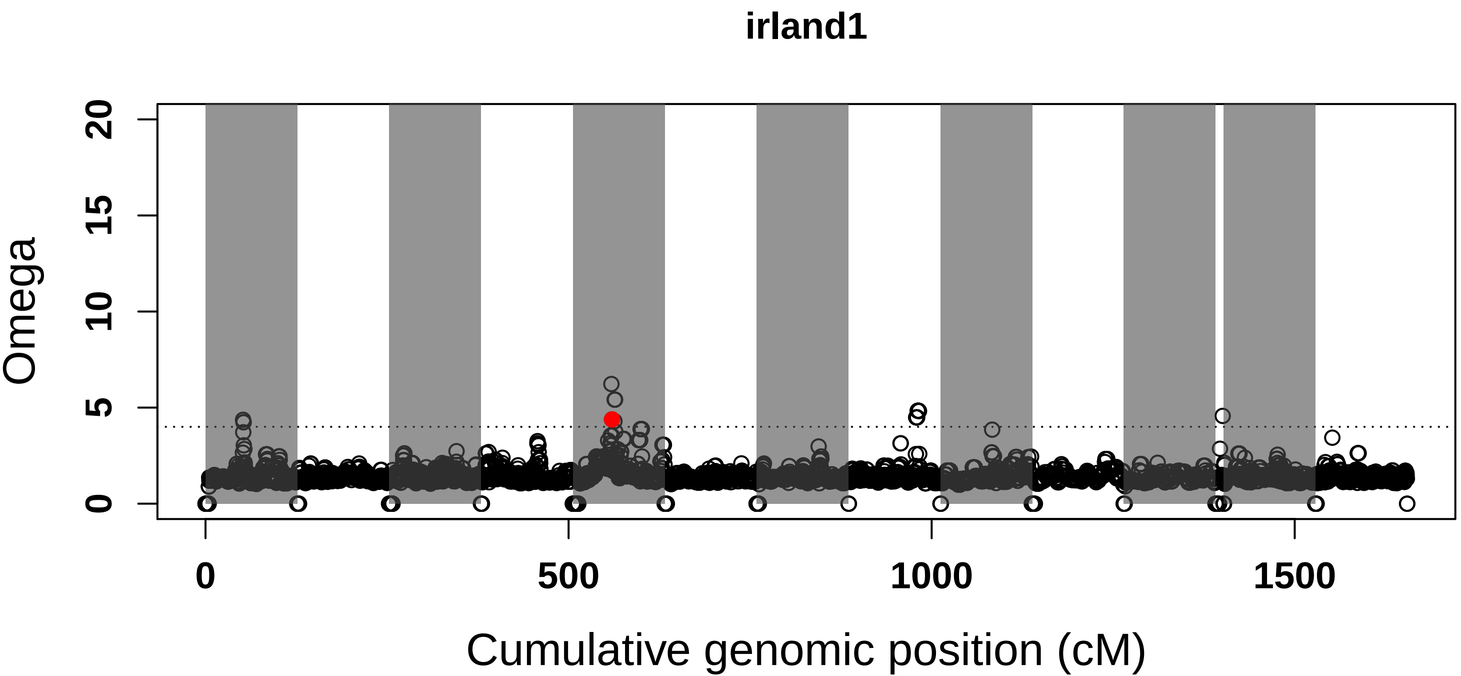
irland1
Omega
0
5
10
15
20
0
500
1000
1500
Cumulative genomic position (cM)

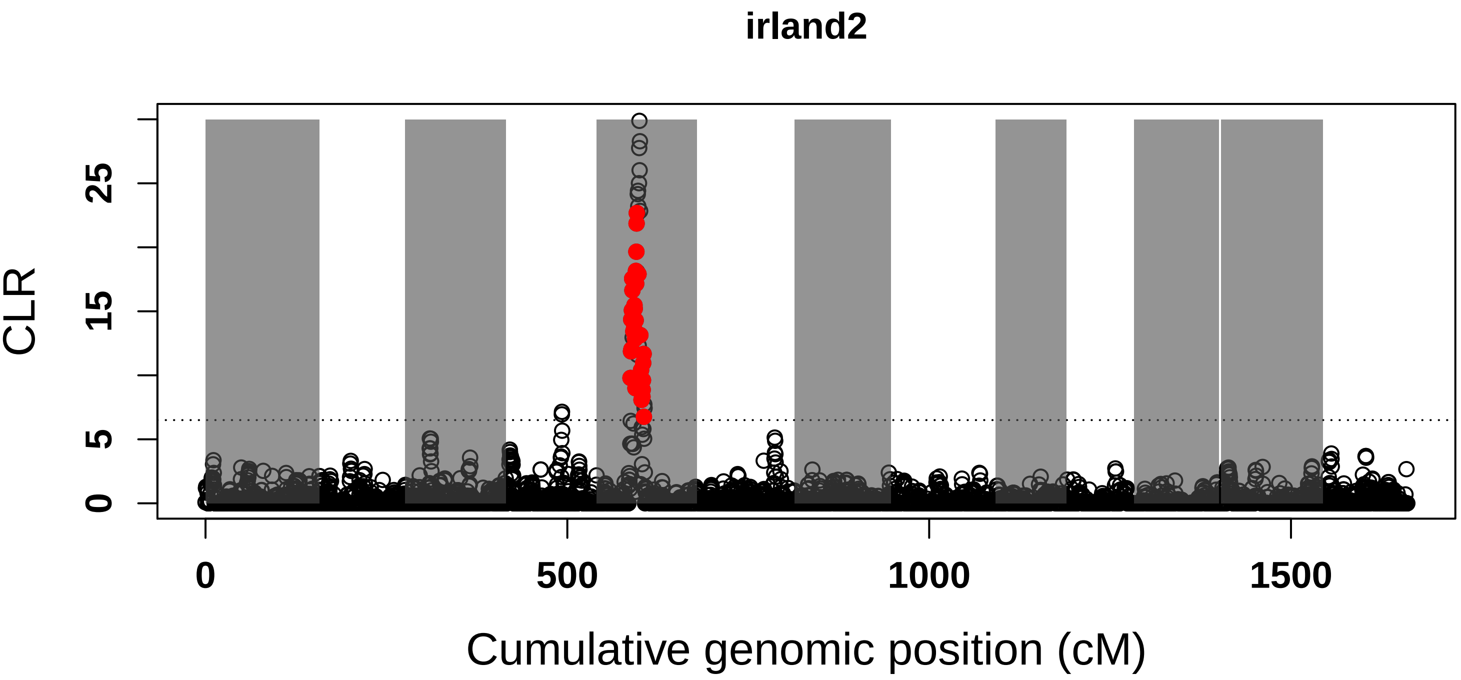
irland2
CLR
0
5
15
25
0
500
1000
1500
Cumulative genomic position (cM)

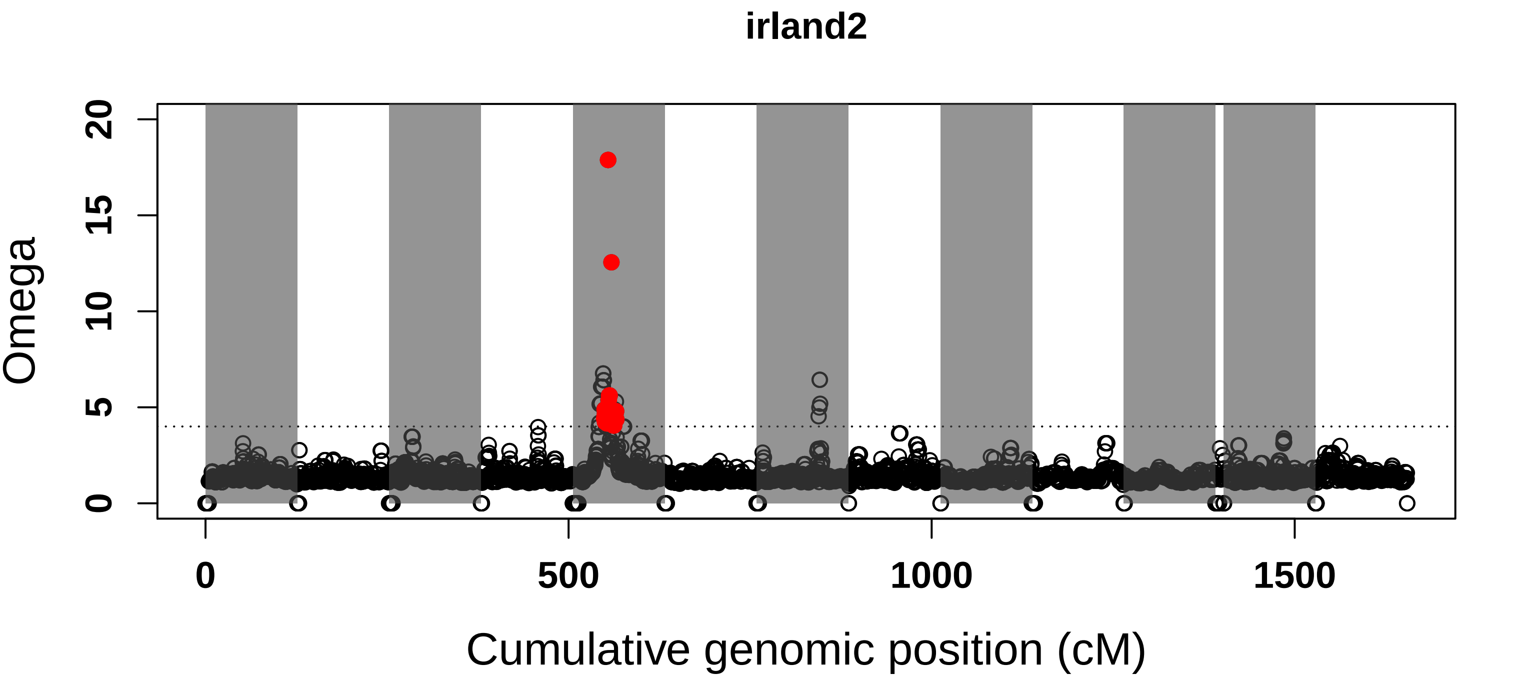

irland2
Omega
0
5
10
15
20
0
500
1000
1500
Cumulative genomic position (cM)

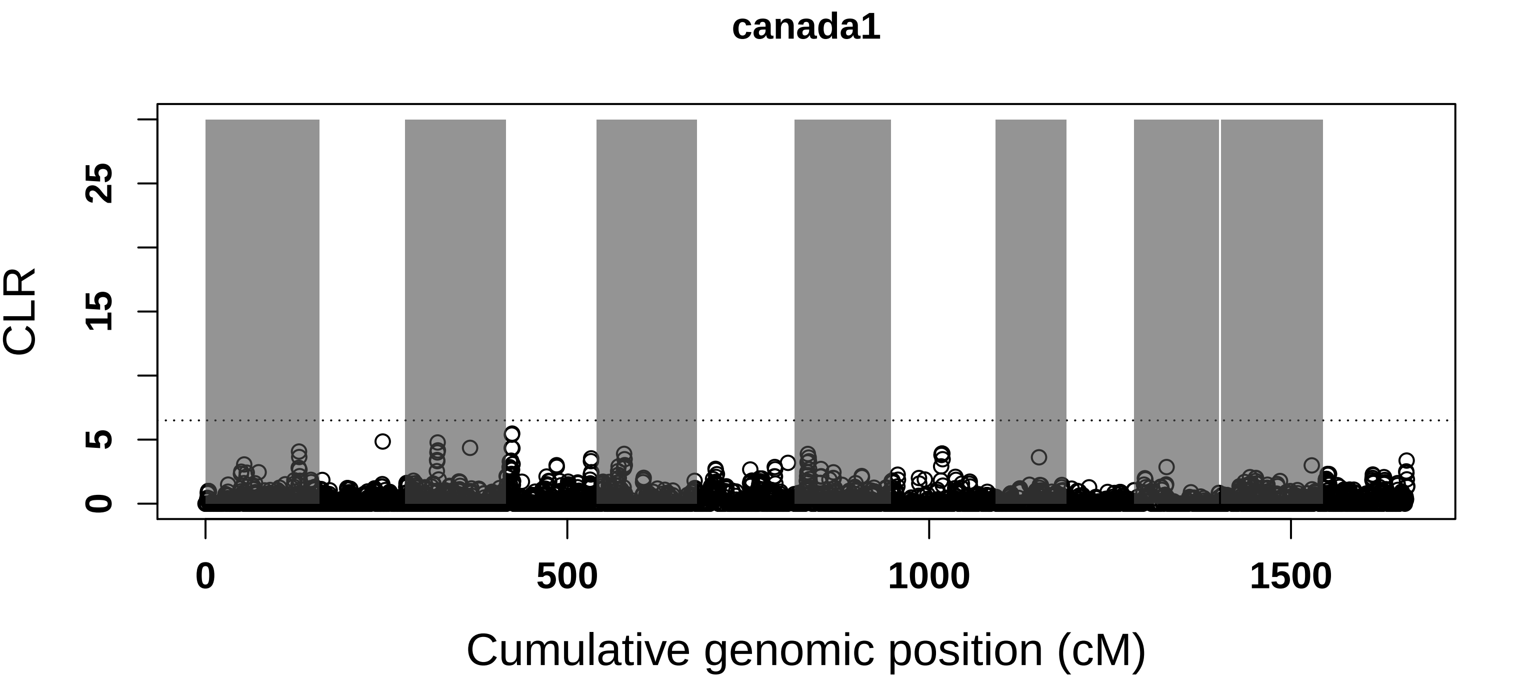

canada1
CLR
0
5
15
25
0
500
1000
1500
Cumulative genomic position (cM)

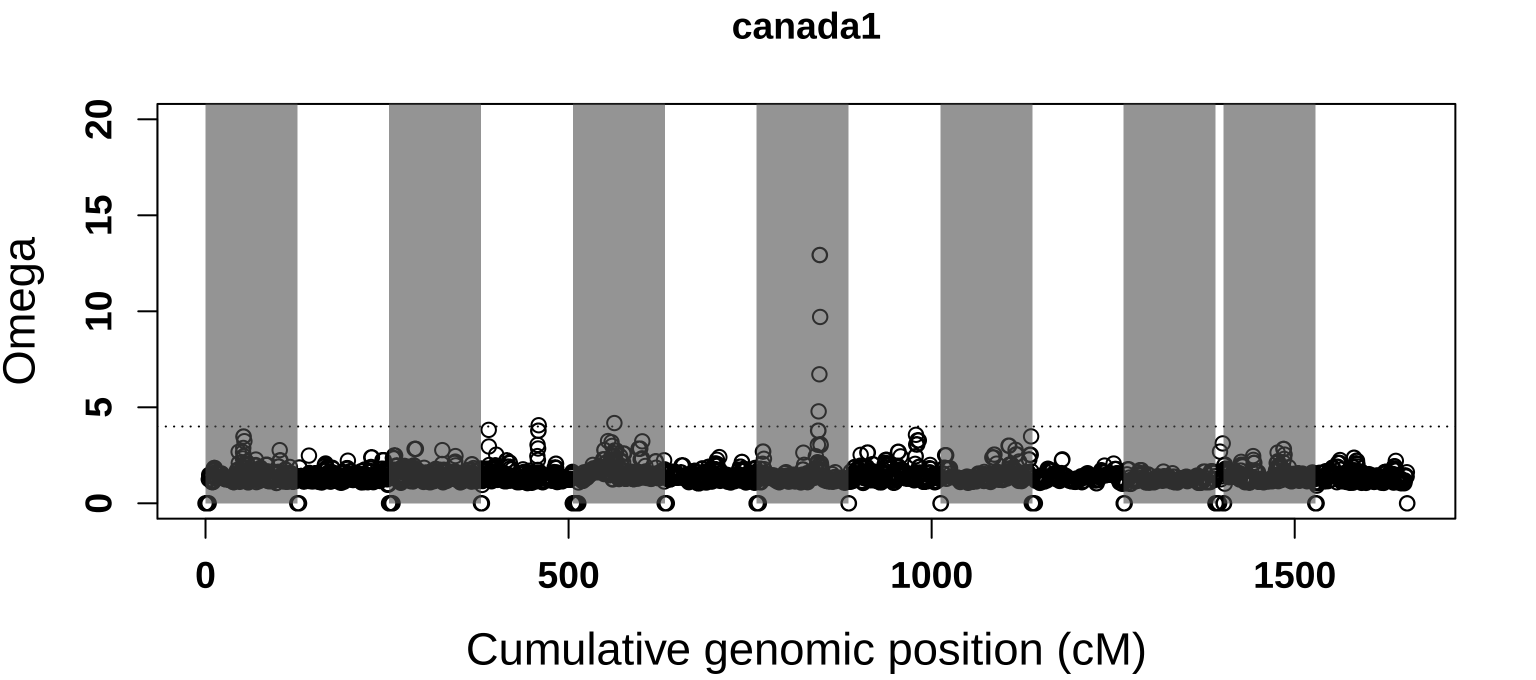

canada1
Omega
0
5
10
15
20
0
500
1000
1500
Cumulative genomic position (cM)

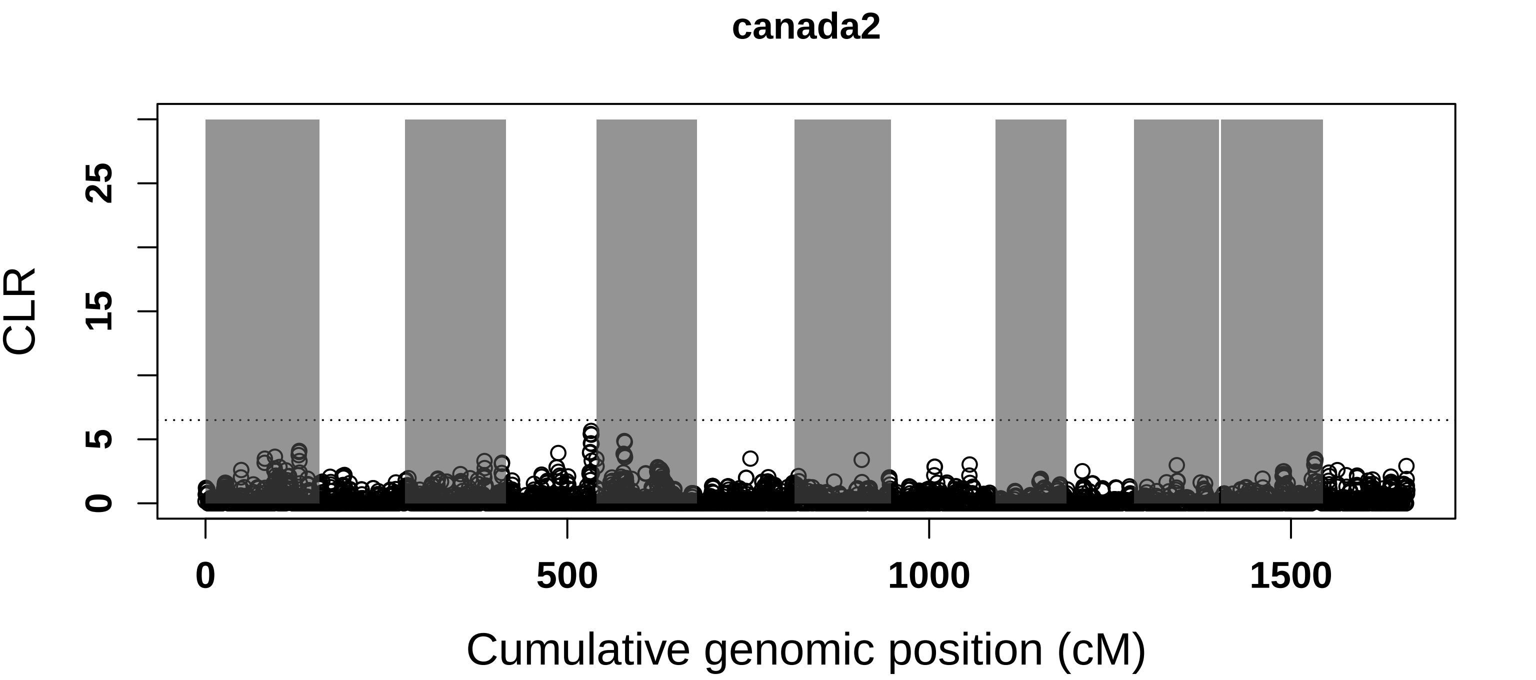
canada2
CLR
0
5
15
25
0
500
1000
1500
Cumulative genomic position (cM)

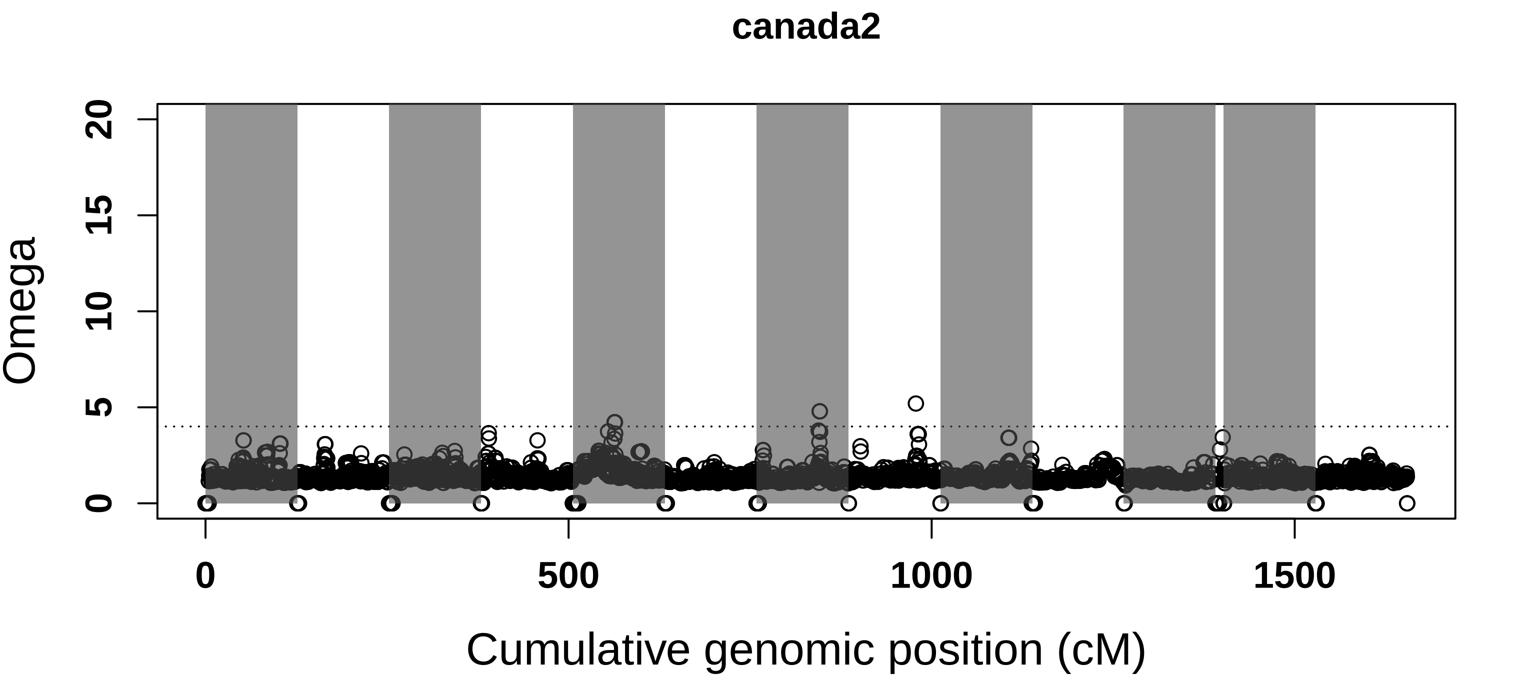

canada2
Omega
0
5
10
15
20
0
500
1000
1500
Cumulative genomic position (cM)
